# A *lil3 chlp* double mutant with exclusive accumulation of geranylgeranyl chlorophyll displays a lethal phenotype in rice

**DOI:** 10.1186/s12870-019-2028-z

**Published:** 2019-10-29

**Authors:** Chunmei Li, Xin Liu, Jihong Pan, Jia Guo, Qian Wang, Congping Chen, Na Li, Kuan Zhang, Bin Yang, Changhui Sun, Xiaojian Deng, Pingrong Wang

**Affiliations:** 1 Rice Research Institute, Sichuan Agricultural University, 211 Huimin Road, Wenjiang District, Chengdu, 611130 China; 2 Zhongkai University of Agriculture and Engineering, 24 Dongsha Street, Haizhu District, Guangzhou, 510225 China

**Keywords:** Chlorophyll biosynthesis, Geranylgeranyl reductase, Leaf-color mutant, Light-harvesting like protein, *LIL3* gene, Phytyl residue, Rice (*Oryza sativa*), Tocopherol biosynthesis

## Abstract

**Background:**

Phytyl residues are the common side chains of chlorophyll (Chl) and tocopherols. Geranylgeranyl reductase (GGR), which is encoded by *CHLP* gene, is responsible for phytyl biosynthesis. The light-harvesting like protein LIL3 was suggested to be required for stability of GGR and protochlorophyllide oxidoreductase in Arabidopsis.

**Results:**

In this study, we isolated a yellow-green leaf mutant, *637ys*, in rice (*Oryza sativa*). The mutant accumulated majority of Chls with unsaturated geranylgeraniol side chains and displayed a yellow-green leaf phenotype through the whole growth period. The development of chloroplasts was suppressed, and the major agronomic traits, especially No. of productive panicles per plant and of spikelets per panicle, dramatically decreased in *637ys*. Besides, the mutant exhibited to be sensitive to light intensity and deficiency of tocopherols without obvious alteration in tocotrienols in leaves and grains. Map-based cloning and complementation experiment demonstrated that a point mutation on the *OsLIL3* gene accounted for the mutant phenotype of *637ys*. *OsLIL3* is mainly expressed in green tissues, and its encoded protein is targeted to the chloroplast. Furthermore, the *637ys 502ys* (*lil3 chlp*) double mutant exclusively accumulated geranylgeranyl Chl and exhibited lethality at the three-leaf stage.

**Conclusions:**

We identified the *OsLIL3* gene through a map-based cloning approach. Meanwhile, we demonstrated that OsLIL3 is of extreme importance to the function of OsGGR, and that the complete replacement of phytyl side chain of chlorophyll by geranylgeranyl chain could be fatal to plant survival in rice.

## Background

Chlorophyll (Chl) is the main component of the photosynthetic pigments, which derives from the esterification of chlorophyllide (Chlide) and phytyl diphosphate (phytyl-PP) [1, 2]. Vitamin E, including tocopherols and tocotrienols, is a potent antioxidant, and is generally believed to arise from the condensation of homogentisic acid (HGA) and phytyl-PP or geranylgeranyl diphosphate (GGPP). Tocopherols carry a phytyl chain, while tocotrienols harbor a geranylgeranyl chain [3, 4]. Chls and tocopherols harbor a common phytyl residue. The phytyl chain of Chls is generated from the reduction of GGPP, while the phytyl chain of tocopherols originates from the reduction of GGPP or Chl degradation [5–8]. The geranylgeranyl reductase (GGR) can either reduce geranylgeranyl Chl (Chl_GG_) into phytyl Chl (Chl_phy_), or reduce GGPP to phytyl-PP to provide phytyl residue for Chl, tocopherol and phylloquinone biosynthesis [5, 9]. Lots of *CHLP* genes encoding GGR have been characterized in photosynthetic bacteria [10–13] and higher plants [5, 9, 14, 15]. In cyanobacterium, *Synechocystis* sp. PCC 6803, the *ΔchlP* mutant exclusively accumulates Chl_GG_ *a* together with low amounts of α-tocotrienol, and displays lethal phenotype in the absence of glucose [16]. In rice (*Oryza sativa*), two *chlp* mutants, *lyl1* and *502ys*, were reported. They exhibited similar yellow-green leaf phenotype and Chl compositions, in which, besides phytyl Chl (Chl_phy_), the majority of Chls were conjugated with incompletely reduced side chains, including Chl_GG_, dihydrogeranylgeranyl Chl (Chl_DHGG_) and tetrahydrogeranylgeranyl Chl (Chl_THGG_) [14, 15].

The light-harvesting complex (LHC) proteins constitute the major outer antenna protein complexes of photosystem I (PSI) and II (PS II). The typical LHC proteins have three transmembrane helices, one or two of which represent LHC motifs because they include potential Chl-binding sites [17]. The LHC-like protein (LIL) contains one to four membrane-spanning domains, and shares one or two of the LHC motifs. Unlike the LHC, LIL does not appear to be involved in light harvesting [18–22]. So far, many LIL proteins have been identified in land plants and green algae as well as in cyanobacteria, including single-helix proteins (OHPs), also known as high-light induced proteins (HLIPs), double-helix stress-enhanced proteins (SEPs), three-helix early light-induced proteins (ELIPs), and four-helix PsbS protein [18, 20–28]. In Arabidopsis, a total of 10 LIL proteins have been characterized. Among these proteins, SEP1, SEP2, LIL3:1 and LIL3:2 belong to the double-helix LIL proteins. SEP1 and SEP2 are stress-induced [23]. LIL3:1 and LIL3:2 contribute to regulating Chl and tocopherol biosynthesis [18, 20].

LIL3 protein has been isolated for the first time from the de-etiolated barley (*Hordeum vulgare*) seedlings [29]. In Arabidopsis, there are two *LIL3* gene copies, *LIL3:1* and *LIL3:2*. Both *lil3:1* and *lil3:2* transposon insertion mutants accumulated a minor fraction of Chls with incompletely reduced side chains, and exhibited indistinguishable phenotypes from those of wild type. However, the *lil3:1 lil3:2* double mutant, which exhibited yellowish green leaves and retarded growth rate, accumulated a majority of Chl_GG_, Chl_DHGG_, and Chl_THGG_, and was deficient in α-tocopherol. LIL3 was suggested to be involved in the formation of phytyl chains of Chl and tocopherol by stabilizing GGR [18]. Nevertheless, no *lil3* mutant has been identified so far in the monocotyledonous plants.

In our study, we isolated a yellow-green leaf mutant *637ys* in rice, accumulating Chls with unsaturated side chains. Compared with the wild type, major agronomic traits dramatically decreased and tocopherols in leaves and grains were significantly lower in *637ys*. Map-based cloning and complementation experiments demonstrated that the mutant phenotype of *637ys* was a result of the point mutation of *OsLIL3* (*LOC_Os02g03330*) gene. *OsLIL3* is mainly expressed in green tissues and its encoded protein is localized to the chloroplast. Furthermore, the *637ys 502ys* double mutant exclusively accumulated Chl_GG_ and exhibited lethality at the three-leaf stage, suggesting that the complete replacement of phytyl side chain of Chl by geranylgeranyl chain could be fatal to plant survival in rice.

## Results

### Isolation and characterization of the *637ys* mutant

**Fig. 1:**
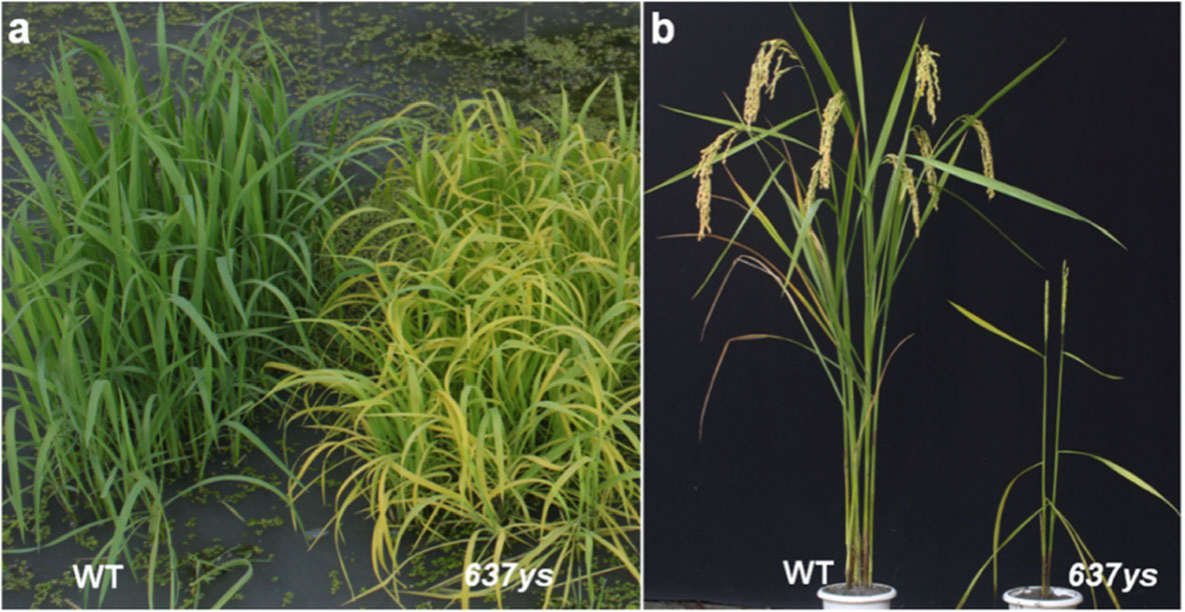
Plant phenotype of the *637ys* mutant and its wild-type ZH11. **a** Four-week-old seedlings. **b** Plants at the grain-filling stage

In a previous study, we isolated the yellow-green leaf mutant *502ys* from *japonica* cultivar Nipponbare (NP), which accumulated the Chls with unsaturated side chains, and was resulted from a point mutation causing an amino acid substitution G206S in *OsCHLP* (*LOC_Os02g51080*) gene [15]. Here, we obtained a new mutant *637ys* from *japonica* cultivar ZH11 via EMS mutagenesis, which accumulated majority of Chls with unsaturated side chains as well as a small amount of Chl_phy_ (about 10% of Chl_phy_ in the wild type) (Additional file 1: Figure S1). The *637ys* mutant displayed a yellow-green leaf phenotype through the whole growth period and grew at a very slow rate. The young leaves from leaf sheaths stayed green in *637ys*, but rapidly turned yellow in several days (Fig. 1). Despite 19 days delay to heading compared to wild type ZH11, *637ys* showed dramatic declines in major agronomic traits. For instance, while the wild type plants had an average on 7.2 of productive panicles, *637ys* plants had only one to at most three, decreasing by 84.7%. The other agronomic traits, plant height, panicle length, No. of spikelets per panicle, seed setting rate, and 1000-grain weight declined by 38.6, 28.2, 80.7, 42.7, and 33.8% correspondingly (Table 1).

**Table 1:** Comparison of major agronomic traits between the *637ys* mutant and its wild-type ZH11

Traits	ZH11 (WT)	*637ys*	Compared with WT
Days to heading (d)	75.0 ± 0.8	94.0 ± 2.2	+ 25.3%^*^
Plant height (cm)	108.2 ± 1.6	66.4 ± 3.5	−38.6%^*^
No. of productive panicles per plant	7.2 ± 0.5	1.1 ± 0.1	−84.7%^*^
Length of main panicle (cm)	23.8 ± 0.8	17.1 ± 1.0	−28.2%^*^
No. of spikelets per panicle	228.9 ± 6.1	44.2 ± 4.1	−80.7%^*^
Seed setting rate (%)	93.0 ± 1.2	50.3 ± 2.8	−42.7%^*^
1000-grain weight (g)	26.9 ± 0.4	17.8 ± 1.0	−33.8%^*^

^*^Significantly different at *P* = 0.05

**Fig. 2:**
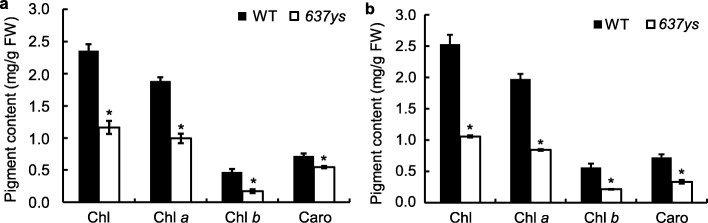
Pigment contents in leaves of the *637ys* mutant and its wild-type ZH11 at different growth stages, in mg g fresh weight^− 1^. **a** Pigment contents at seedling stage. **b** Pigment contents at heading stage. Data are shown as mean ± SD. Error bars represent standard deviations of three independent biological replicates. Asterisks indicate statistically significant differences (with Student’s *t* test) compared with the wild-type at *P* < 0.01

To quantify the mutant phenotype of *637ys*, we determined the contents of photosynthetic pigments in the *637ys* and ZH11 plants at both seedling and heading stages. The amount of total Chl, Chl *a*, Chl *b*, and Caro in *637ys* remarkably decreased by 50.6 to 58.2%, 47.4 to 57.3%, 63.4 to 61.4%, and 24.0 to 53.4%, respectively, compared to those in wild type (Fig. 2). These results suggested that the yellow-green leaf phenotype was due to its decreased level of photosynthetic pigments.

**Fig. 3:**
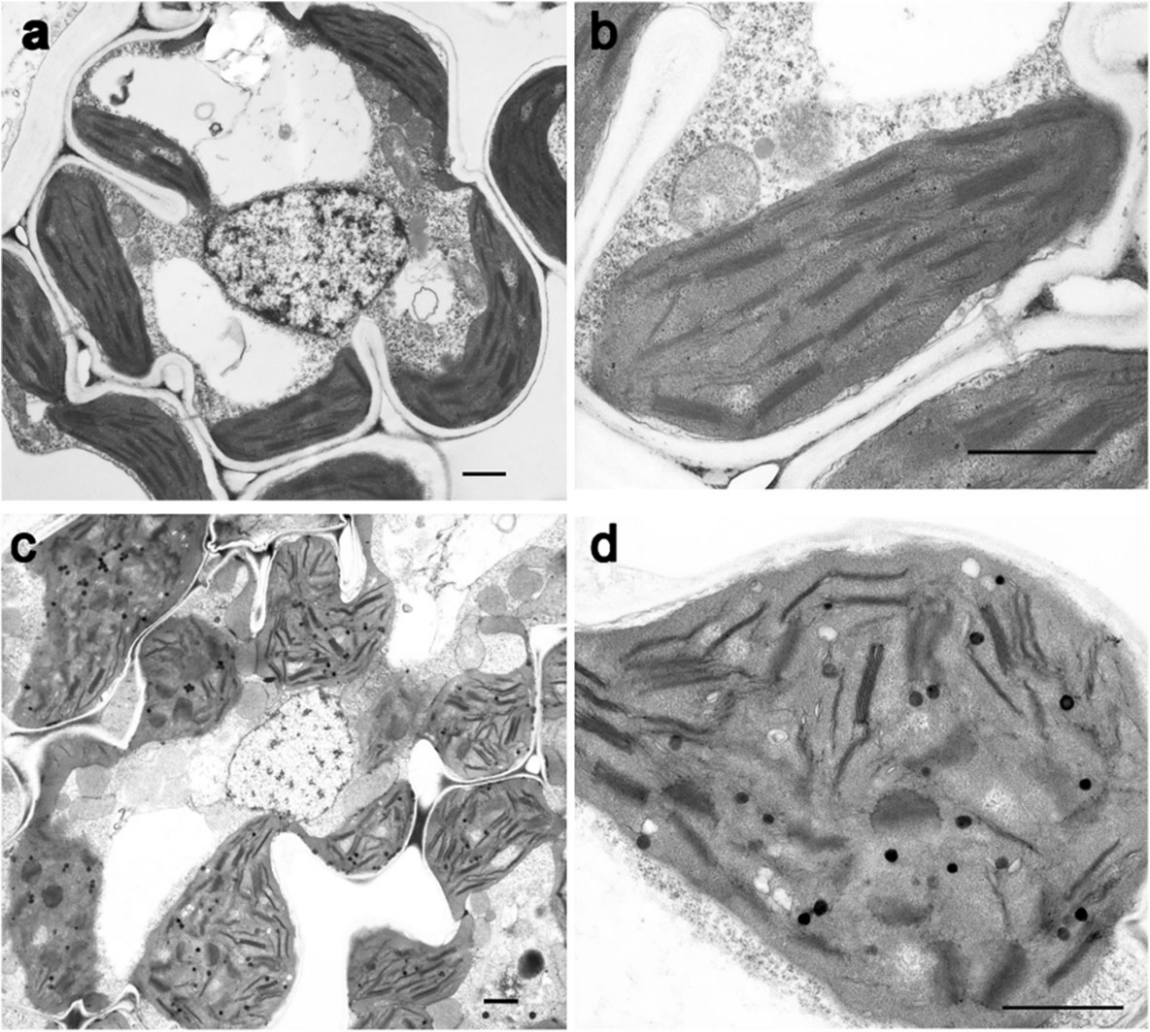
Ultrastructure of mesophyll cells in the *637ys* mutant and its wild-type ZH11 under transmission electron microscopy. **a** and **b** Mesophyll cells and chloroplasts of ZH11, respectively. **c** and **d** Mesophyll cells and chloroplasts of *637ys*, respectively. Bars = 1 μm

To explore if the reduced contents of photosynthetic pigments affect the development of chloroplasts in *637ys*, we investigated the ultrastructure of chloroplasts under transmission electron microscopy. A number of grana stacks consisting of well-developed grana lamellae connected by stroma lamellae were present in wild type chloroplasts (Fig. 3a, b). However, the chloroplasts were swollen in *637ys.* Even if some grana stacks existed, the grana lamellae were less densely spaced than those in wild type and changed into disarray arrangement. Furthermore, stroma density decreased and osmiophilic globules occurred in the stroma in *637ys* (Fig. 3c, d). These results revealed that the development of chloroplast was suppressed in the *637ys* mutant.

### Sensitivity of *637ys* mutant to temperature and light intensity

**Fig. 4:**
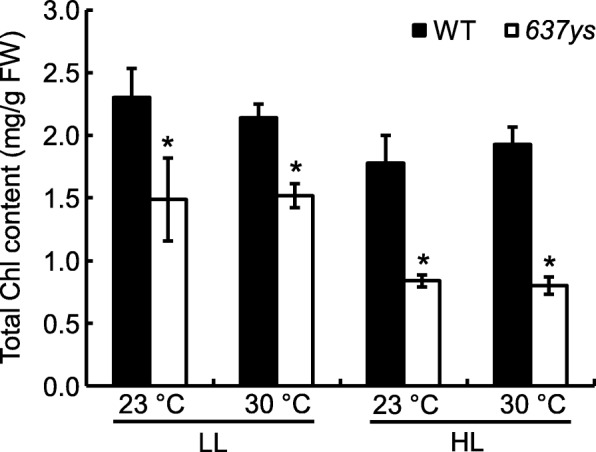
Total Chl contents in *637ys* and its wild-type (WT) grown under low light (LL) or high light (HL) at constant temperature (23 °C or 30 °C), in mg g fresh weight^− 1^. Data are shown as mean ± SD. Error bars represent standard deviations of three independent biological replicates. Asterisks indicate statistically significant differences (with Student’s *t* test) compared with the wild-type at *P* < 0.01

To investigate if the mutant phenotype was dependent upon temperature, the *637ys* and wild type plants grown in the growth chamber were treated by two different temperature conditions (constant 23 °C and 30 °C). As a consequence, the *637ys* mutant grown under different temperature conditions exhibited indistinguishable leaf-color phenotype (Additional file 2: Figure S2 a1, a2). Its Chl contents significantly reduced, compared to wild type, but there was no obvious difference between low temperature and high temperature, which was similar to those in its wild type (Fig. 4; Additional file 3: Table S1; Additional file 4: Table S2). These data suggested that the phenotype of *637ys* was independent upon temperature.

All mutants accumulating the Chls with unsaturated side chains displayed sensitivity to light intensity [14, 30]. Correspondingly, the phenotype of *637ys* mutant under low light (80 μmol m^− 2^ s^− 1^) and high light (300 μmol m^− 2^ s^− 1^) was also investigated. The mutant displayed yellow-green leaf phenotype under high light condition (Additional file 2: Figure S2 a1-b2). Meanwhile, its Chl contents significantly declined, compared to that under low light condition, while the Chl contents in wild type remained relatively stable (Fig. 4; Additional file 5: Table S3). These data suggested that the phenotype of *637ys* depended on light intensity.

### Analysis of vitamin E in leaves and grains

**Fig. 5:**
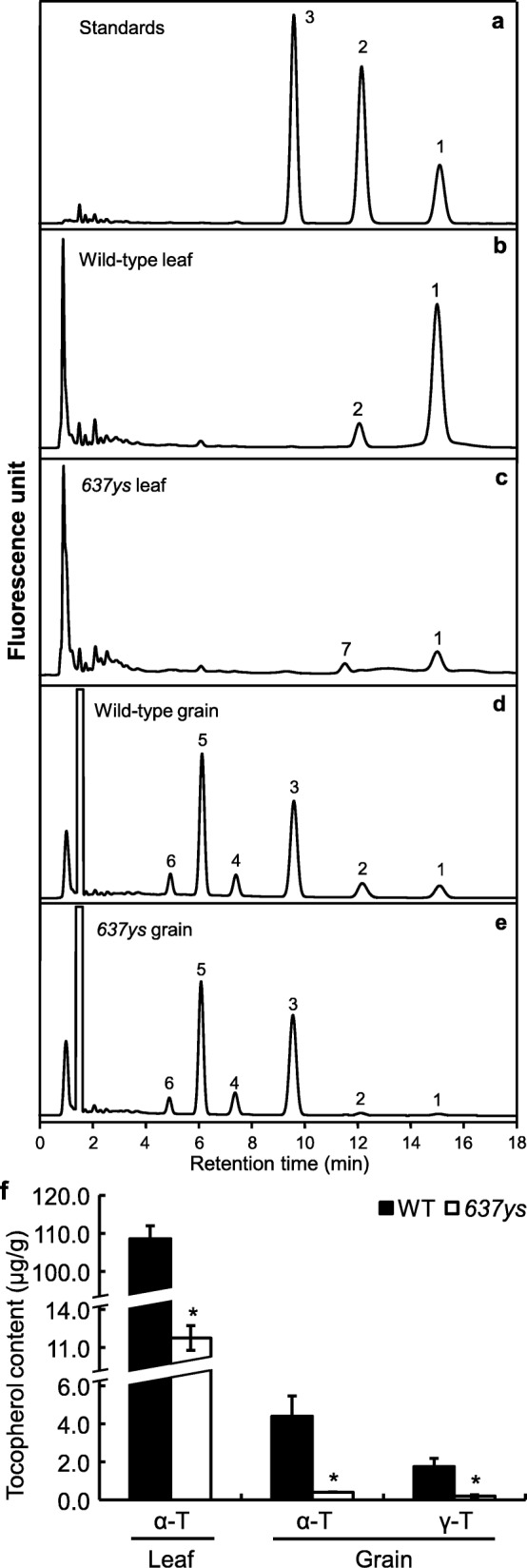
Analysis of vitamin E in leaves and grains of *637ys*. Elution profiles of the tocopherol standards (**a**), tocopherols in leaves of wild-type ZH11 (**b**) and *637ys* (**c**), tocopherols and tocotrienols in grains of ZH11 (**d**) and *637ys* (**e**) were detected by fluorescence with excitation at 290 nm and emission at 330 nm. Tocopherol contents in leaves and grains of ZH11 and *637ys* (**f**) were quantified by using tocopherol standards. α-T, α-tocopherol; γ-T, γ-tocopherol. The tocopherol standards were prepared by pooling identical volumes of α-tocopherol, γ-tocopherol and δ-tocopherol at the same concentration. Peaks 1 and 2 represent α-tocopherol and γ-tocopherol; Peak 3 is δ-tocopherol which does not exist in rice and was used as control. Peaks 4, 5 and 6 represent α-tocotrienol, γ-tocotrienol and δ-tocotrienol, respectively. Peak 7 might be the isomer of γ-tocopherol. Error bars represent standard errors of three independent biological replicates. Asterisks indicate statistically significant differences (with Student’s *t* test) compared with the wild-type at *P* < 0.01

Tocopherols and tocotrienols constitute vitamin E. The phytyl-PP forms the side chains of both Chl_phy_ and tocopherols, and the GGPP forms the side chains of Chl_GG_ and tocotrienols [4, 5]. Because of the accumulation of Chls with unsaturated side chains in *637ys* mutant, to investigate whether the composition of vitamin E was affected, we analyzed the tocopherol and tocotrienol compositions in leaves and grains in *637ys* and its wild type ZH11 by HPLC. In leaves, HPLC profiles of vitamin E showed that α-tocopherol was abundant in wild type, while the elution peak of α-tocopherol in *637ys* was much lower than that in wild type and significantly decreased by 89.2% (peak 1 in Fig. 5b, c; Fig. 5f). At the same time, a small amount of γ-tocopherol was detected in the wild type leaves (peak 2 in Fig. 5b), but not in the *637ys* mutant. It is noteworthy that a minor peak (peak 7 in Fig. 5c), whose retention time was 0.5 min fewer than the peak of γ-tocopherol in wild type, was detected in the *637ys* mutant. We speculated that the minor peak in the *637ys* was likely to be an isomer of γ-tocopherol [4]. In addition, tocotrienols in leaves were almost undetectable either in wild type or *637ys* mutant (Fig. 5b, c). In grains, HPLC analysis showed that the *637ys* mutant had few of α-tocopherol or γ-tocopherol declining by 90.9 and 89.7% respectively (peaks 1 and 2 in Fig. 5d, e; Fig. 5f), but considerable level of tocotrienols comparable to ZH11 (peaks 4, 5 and 6 in Fig. 5d, e; Fig. 5f; Additional file 6: Figure S3) [4, 14]. These results indicated that tocopherols were deficient, but the accumulation of tocotrienols was not affected in *637ys*.

### Map-based cloning of the *637ys* mutant gene

We crossed *637ys* with *502ys* (*chlp*) mutant, and the resulting F_1_ plants all displayed normal green phenotype, which indicated that *637ys* and *502ys* mutant genes are not allelic. In order to genetically analyze the *637ys* mutant, we crossed *637ys* with its wild type ZH11 and normal green *indica* cultivar G46B. All resulting F_1_ plants exhibited a normal green phenotype. Leaf-color phenotypes of the F_2_ populations segregated with a ratio of 3:1 (χ^2^ < χ^2^_0.05_ = 3.84, *P* > 0.05), suggesting that a single recessive gene was responsible for the yellow-green leaf phenotype of *637ys*.

**Fig. 6:**
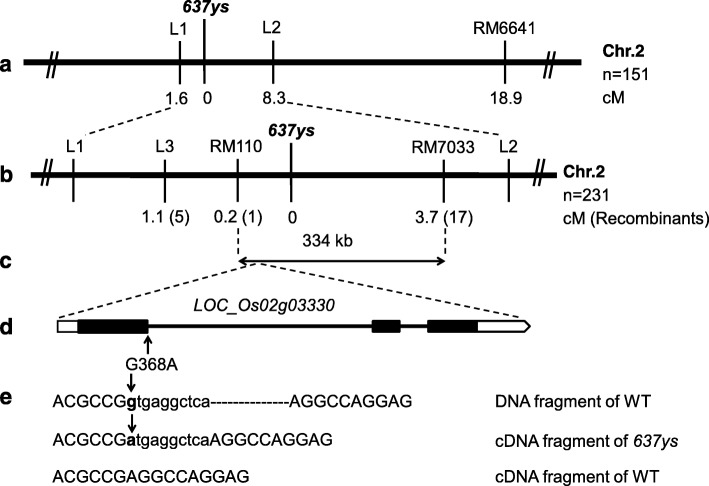
Map-based cloning of the *637ys* locus. **a** The *637ys* locus was mapped to a region between InDel markers L1 and L2 on the short arm of the chromosome 2 (Chr.2). **b** The *637ys* locus was narrowed down to a region between SSR markers RM110 and RM7033 using 231 F_2_ plants. **c** The 334 kb-region contains 62 putative genes. **d** The candidate *LOC_Os02g03330* gene is constituted by three exons and two introns, and G-to-A substitution occurred at the first nucleotide of the first intron in the *637ys* mutant, the position 368 of its coding region. **e** 10 bp of intron sequence inserted in the cDNA sequence of *OsLIL3* in the *637ys* mutant. The sequences of intron and exon are shown in lower and upper case, respectively

Next, the F_2_ population from the cross between *637ys* and G46B was constructed for mapping. Preliminary mapping results suggested that the *637ys* locus was linked with the SSR marker RM6641 on the short arm of Chromosome 2, and then we used 2 SSR markers and 3 InDel markers (Additional file 7: Table S4) to locate *637ys* in a 334-kb region between SSR markers RM110 and RM7033 with 0.2 and 3.7 cM respectively (Fig. 6a, b). Within this region, we have further developed a total of 8 InDel and SSR markers which however showed no polymorphism between *637ys* and G46B.

Although there are 62 putative genes within the 334-kb region according to the Rice Genome Annotation Project (http://rice.plantbiology.msu.edu/annotation_pseudo_current.shtml), the genetic distance between the *637ys* locus and RM110 is much shorter than that between *637ys* and RM7033, which suggested that the *637ys* locus should be close to RM110 (Fig. 6b, c). Therefore we analyzed the genes in this region starting from RM110 based on the annotations and the TargetP and ChloroP (http://www.cbs.dtu.dk/services/TargetP/; http://www.cbs.dtu.dk/services/ChloroP/) [31, 32]. Of particular interest, we found a gene, *LOC_Os02g03330*, which encodes a light-harvesting like protein with 57 and 58% identities to LIL3:1 and LIL3:2 in Arabidopsis respectively (Fig. 6d). The *lil3:1 lil3:2* double mutant of Arabidopsis accumulated Chl_GG_, Chl_DHGG_, and Chl_THGG_, showing a similar phenotype to the *637ys* mutant [18]. Then we sequenced the DNA extracted from *637ys* mutant and its wild type, and the results revealed that a G-to-A substitution occurred in position 368 of the gene (Fig. 6d). Furthermore, we sequenced cDNA of *LOC_Os02g03330* in *637ys* mutant and ZH11. Sequence alignment showed that the substitution occurred at the first nucleotide of the first intron in the DNA sequence of *LOC_Os02g03330*, and consequently caused 10 bp-intron sequence insertion in cDNA sequence of this gene in *637ys* mutant (Fig. 6e). Thereby the reading frame shift in *LOC_Os02g03330* resulted in premature translation of its encoded protein in *637ys* (Additional file 8: Figure S4a). Therefore, *LOC_Os02g03330* was considered as the candidate gene of *637ys* mutant, and designated as *OsLIL3.*

**Fig. 7:**
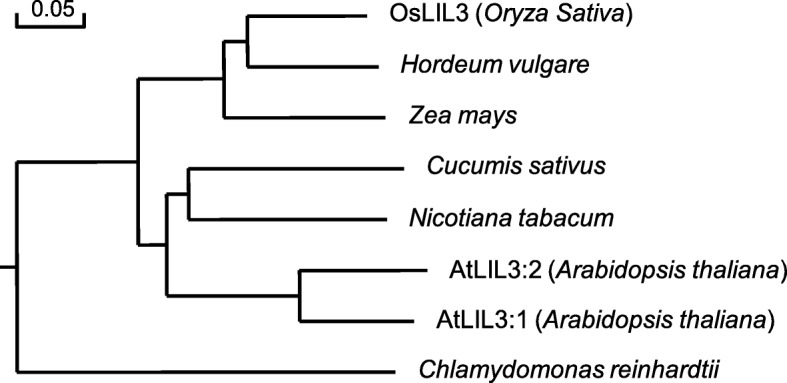
Phylogenetic tree of OsLIL3 and its homologues. The rooted tree using percentage identities is based on a multiple sequence alignment generated with the program DNAMAN. Scale bar represents percentage substitution per site. GenBank accession numbers for the respective protein sequences are as follows: *Oryza sativa* OsLIL3 (LOC_Os02g03330); *Hordeum vulgare* (BAJ88054.1); *Zea mays* (NP_001167647.1); *Cucumis sativus* (XP_004147237.1); *Nicotiana tabacum* (XP_016470950.1); *Arabidopsis thaliana* AtLIL3:1 (NP_567532.1, At4g17600) and AtLIL3:2 (NP_199522.2, At5g47110); *Chlamydomonas reinhardtii* (XP_001699421.1)

Searching in the rice genome database revealed that *OsLIL3* is a single copy gene. Alignment of sequenced DNA and cDNA showed *OsLIL3* has three exons and two introns and its full length of genomic sequence and cDNA are 2384 bp and 753 bp, respectively. The protein encoded by *OsLIL3* comprises 250 of amino acids, which has a molecular weight of 27.6 kDa. The OsLIL3 contains a predicted chloroplast transit peptide of 44 amino acids at N-terminus (Additional file 8: Figure S4) [31, 32]. The structure-prediction programs TMHMM and HMMTOP (http://www.cbs.dtu.dk/services/TMHMM/; http://www.enzim.hu/hmmtop/html/submit.html) [33, 34] indicate two transmembrane helices, among which the first helix of OsLIL3 proteins includes the well-conserved LHC motif (Additional file 9: Figure S5). According to multiple alignment of OsLIL3 and its homologues in different species, OsLIL3 has a high similarity to its homologues in monocotyledonous plants, barley (*Hordeum vulgare*), and maize (*Zea mays*) and dicotyledonous plants, cucumber (*Cucumis sativus*) and tobacco (*Nicotiana tabacum*), with 72, 72, 70 and 63% respectively. Phylogenetic analysis revealed that OsLIL3 is more closely related to its homologues from barley and maize than those from other species (Fig. 7).

### Complementation of the *637ys* mutant

**Fig. 8:**
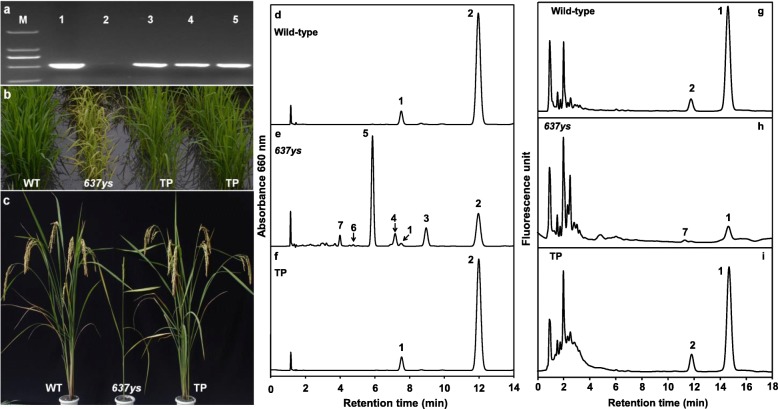
Complementation of the *637ys* mutant with *OsLIL3* gene. **a** Identification of transgenic plants by PCR. M, DL 2000 marker; 1, pC2300-*OsLIL3* plasmid (positive control); 2, *637ys* (negative control); 3–5, positive transgenic plants. **b** and **c** Phenotypes of wild-type (WT), *637ys* and PCR-positive transgenic plants (TP) at the seedling stage and at the grain-filling stage, respectively. **d**, **e** and **f** The elution profiles of Chl in leaves of WT, *637y*, and TP at the seedling stage were detected at 660 nm, respectively. Peaks 2, 3, 4, and 5 represent Chl_phy_ *a*, Chl_THGG_ *a*, Chl_DHGG_ *a*, and Chl_GG_ *a*, respectively. Peaks 1, 6 and 7 represent Chl_phy_ *b*, Chl_DHGG_ *b*, and Chl_GG_ *b*, respectively. The absorption spectra of elution profiles in acetone are the same as those in Additional file 1: Figure S1c and d. **g**, **h** and **i** The elution profiles of the tocopherols in leaves of WT, *637ys*, and TP at the seedling stage were detected, respectively, by fluorescence with excitation at 290 nm and emission at 330 nm. Peaks 1 and 2 represent α-tocopherol and γ-tocopherol, respectively, and Peak 7 might be the isomer of γ-tocopherol

To confirm that the mutation of *OsLIL3* caused the yellow-green leaf phenotype in *637ys* mutant, we performed a complementation assay. The construct pC2300-*OsLIL3* carrying *OsLIL3* driven by rice *Actin1* promoter was generated by inserting full length cDNA of *OsLIL3* into pCAMBIA2300 vector. Then the final construct was transformed into *637ys* mutant mediated by *Agrobacterium*, and 11 transgenic lines were identified as positive transgenic plants which recovered to normal green (Fig. 8a–c). In addition, we determined the Chl compositions in these positive lines. As shown in Fig. 8d–f, positive transgenic lines only accumulated Chl_phy_ instead of Chl conjugated with unsaturated geranylgeraniol side chains. Meanwhile, their levels of tocopherols in leaves reached to those of wild type (Fig. 8g–i). These data suggested that the *OsLIL3* gene rescued the deficiency of Chl_phy_ and tocopherols in *637ys*, from which we conclude that the mutant phenotype of *637ys* was due to the single base pair mutation in the *OsLIL3* gene.

### Subcellular localization of OsLIL3 protein

**Fig. 9:**
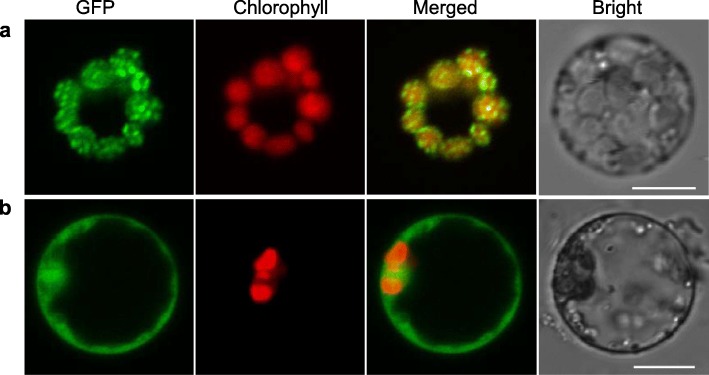
Subcellular localization of OsLIL3 protein. Fluorescence signals were visualized using a laser-scanning confocal microscopy. Green fluorescence shows GFP, red fluorescence indicates chloroplast autofluorescence, yellow fluorescence indicates images with the two types of fluorescence merged, and bright field images show rice protoplasts. **a** GFP signals of the OsLIL3-GFP fusion protein. **b** Empty GFP vector without a specific targeting sequence. Scale bars = 10 μm

OsLIL3 was predicted to contain a chloroplast transit peptide with 44 amino acid residues at its N-terminus by using TargetP and ChloroP (Additional file 8: Figure S4) [31, 32]. In order to prove this prediction, we generated constructs expressing OsLIL3-green fluorescent protein (GFP) fusion protein, pCAMBIA2300–35 s-*OsLIL3*-*GFP*, transformed rice protoplasts with the final construct and pCAMBIA2300–35 s-*GFP* (as control) respectively, and observed transformed protoplasts under a laser-scanning confocal microscopy. In accordance with what was predicted by TargetP and ChloroP, the green fluorescence of OsLIL3-GFP fusion protein overlapped with the red autofluorescence of Chl in the chloroplasts, while GFP itself was expressed all over the whole cell (Fig. 9). These data provide strong evidence that OsLIL3 is chloroplast targeted.

### Expression pattern of the *OsLIL3* gene

**Fig. 10:**
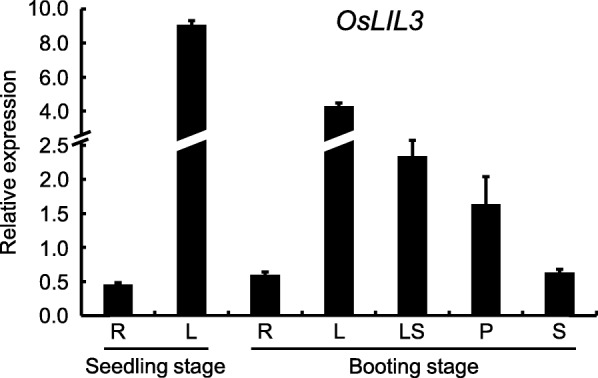
Expression patterns of *OsLIL3* gene. The expression levels were determined by real-time PCR in root (R), and leaf (L), leaf sheath (LS), panicle (P), and stem (S) of wild type grown in a paddy field. The levels of transcripts were normalized to rice *Actin 1* gene as an internal control

To investigate where the *OsLIL3* gene was expressed, we analyzed its level of transcripts in different tissues by qRT-PCR, including roots and leaf blades at seedling stage, stems, leaf blades, leaf sheaths, and young panicles of the wild type at both seedling stage and booting stage. The results demonstrated that the *OsLIL3* was differentially expressed in different tissues. Specifically, leaf blades ranked the first, with leaf sheaths and young panicles followed, while stems and roots had relatively low levels of transcripts (Fig. 10). The results indicated that *OsLIL3* was mainly expressed in green tissues.

### Expression analysis of genes at seedling stage for photosynthesis and Chl synthesis

**Fig. 11:**
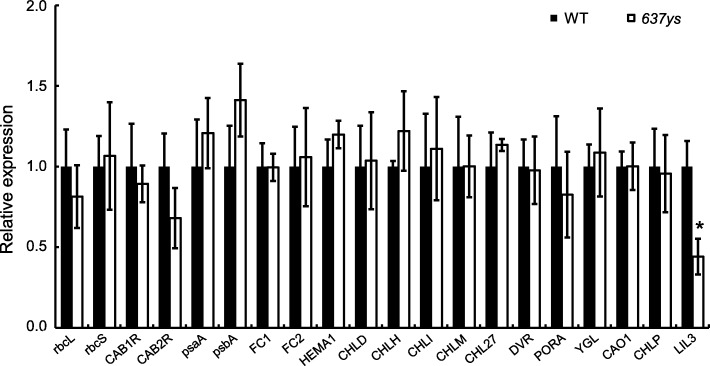
Expression analysis of genes involved in photosynthesis and Chl biosynthesis between the *637ys* mutant and its wild-type ZH11. *Actin 1* was amplified as an internal reference. The expression level of each gene in wild types was set to 1.0, and those in *637ys* mutant were calculated accordingly. Error bars represent standard errors of three independent biological replicates. The asterisk indicates statistically significant differences (with Student’s *t* test) compared with the wild-type at *P* < 0.05

Since the Chl compositions changed in the *637ys* mutant, to investigate whether expressions of the genes associated with photosynthesis and Chl synthesis were affected, we examined transcript levels of 19 related genes and *OsLIL3* at seedling stage in *637ys*. Among these genes, six genes are related to photosynthesis [35], including *rbcL* and *rbcS* (Rubisco large and small subunits, respectively), *CAB1R* and *CAB2R* (Chl *a*/*b*-binding proteins of PS II), *psaA* and *psbA* (two reaction center polypeptides). Two genes are related to the heme branch, including *FC1* and *FC2* (ferrochelatase1 and 2) [36]. Eleven genes encode enzymes involved in Chl biosynthesis, including *HEMA1* (glutamyl-tRNA reductase), *CHLD*, *H* and *I* (D, H and I subunits of Mg chelatase), *CHLM* (Mg-protoporphyrin IX methyltransferase), *CHL27* (Mg-protoporphyrin IX monomethylester cyclase), *DVR* (divinyl reductase), *PORA* (protochlorophyllide oxidoreductase), *OsCHLP*, *YGL* (*CHLG*, Chl synthase), and *CAO1* (chlorophyllide a oxygenase) [2, 14, 15, 35–39]. However, except that *OsLIL3* was significantly down-regulated in the *637ys* mutant, no significant change in transcription levels of the other genes detected was found. Interestingly, the expression of *OsCHLP* was not significantly affected in the *637ys* (Fig. 11). The results suggested that the functional defect in OsLIL3 did not affect the transcriptional levels of the aforementioned genes for photosynthesis and Chl and heme synthesis, which were consistent with those from *lil3:1* and *lil3:2* single mutants and the *lil3:1 lil3:2* double mutant [20].

### Phenotype and Chl composition of the *502ys 637ys* double mutant

**Fig. 12:**
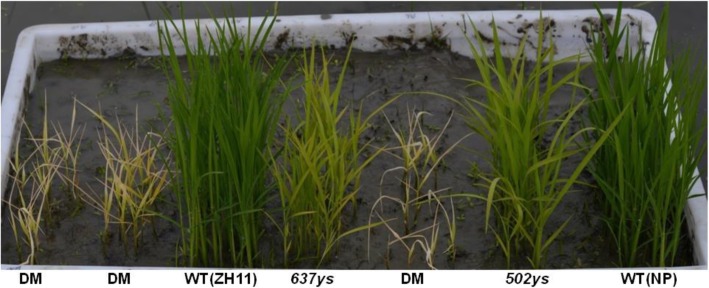
Phenotypic comparison of the wild-type ZH11 and Nipponbare (NP), the *637ys* mutant, the *502ys* mutant and the *637ys 502ys* double mutant (DM) at the three-leaf stage. DM was obtained in the F_2_ population derived from the cross between *637ys* and *502ys*, and the DM plants were confirmed by PCR and sequencing. To avoid competition for light with other plants stronger than DM plants in the F_2_ population, only homozygous DM plants were retained in the soil

**Fig. 13:**
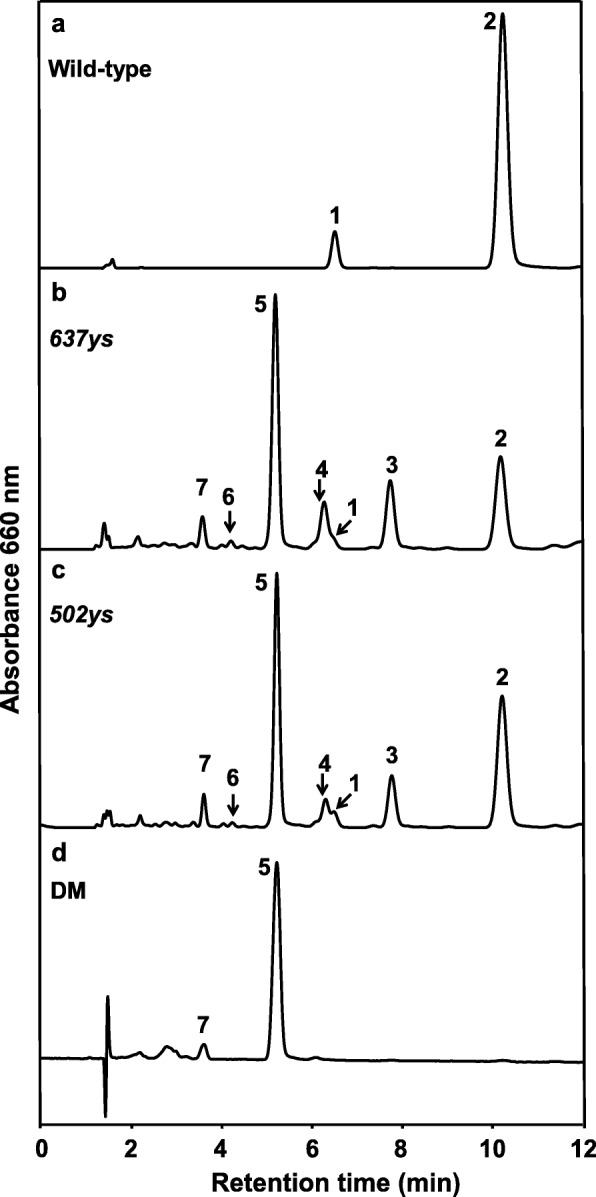
Chl composition analysis of the wild-type, the *637ys* mutant, the *502ys* mutant and the *637ys 502ys* double mutant (DM). The elution profiles of Chls in wild-type ZH11 and Nipponbare (**a**), *637ys* (**b**), *502ys* (**c**) and DM (**d**) were detected at 660 nm by using HPLC. Peaks 2, 3, 4, and 5 represent Chl_phy_ *a*, Chl_THGG_ *a*, Chl_DHGG_ *a*, and Chl_GG_ *a*, respectively. Peaks 1, 6 and 7 represent Chl_phy_ *b*, Chl_DHGG_ *b*, and Chl_GG_ *b*, respectively. The absorption spectra of elution profiles in acetone are the same as those in Additional file 1: Figure S1c and d

Both *637ys* and *502ys* accumulated Chls with unsaturated side chains and defected in tocopherols [14, 15]. To further explore the association between *OsCHLP* and *OsLIL3* in Chl phytyl biosynthesis in rice, we generated the homozygous *637ys 502ys* double mutant by crossing *502ys* mutant with *637ys* mutant. Under natural sunlight condition, all F_1_ plants displayed a normal green phenotype. Then, F_2_ plants displayed a segregation of four phenotypic classes at the early seedling stage: normal green plants, yellow-green plants similar to *502ys*, yellow plants similar to *637ys*, and more severely yellow and smaller plants which were eventually confirmed as *637ys 502ys* double mutants by sequencing the mutation sites of *OsCHLP* in *502ys* and *OsLIL3* in *637ys* (Fig. 12). To avoid competition for light with other plants stronger than double mutants in the F_2_ population, only *637ys 502ys* double mutant plants were retained in the soil. Unlike *502ys* or *637ys* (Fig. 13b, c), only Chl_GG_ *a* and Chl_GG_ *b* were exclusively accumulated, and none of Chl_DHGG_, Chl_THGG_, or Chl_phy_ was detectable in the double mutants by HPLC (Fig. 13d). To investigate the chloroplast development in the double mutants, we also observed the ultrastructure of chloroplasts under transmission electron microscopy. Compared to *637ys* and *502ys* (Fig. 3) [15], few of well-developed grana stacks existed in the double mutant (Additional file 10: Figure S6). Unfortunately, the double mutants all died at the three-leaf stage (Fig. 12). In addition, we also investigated the *637ys 502ys* double mutant grown in a growth chamber under low light at constant 23 °C, and obtained similar results of exclusive accumulation of Chl_GG_ and lethal phenotype at the three-leaf stage (Additional file 11: Figure S7). These results suggested that the complete absence of Chl_phy_ or only the presence of Chl_GG_ in the double mutant could be fatal to rice seedling.

## Discussion

LIL3 protein was firstly isolated from pigment-binding complexes in de-etiolated barley seedlings [29]. The Arabidopsis genome harbors two *LIL3* gene copies, *LIL3:1* and *LIL3:2*. Both *lil3:1* and *lil3:2* transposon insertion mutants accumulated a majority of Chl_phy_ along with tiny amounts of Chls with incompletely reduced side chains [18]. However, no *lil3* mutant has been identified in the monocotyledonous plants. In our study, we isolated a yellow-green leaf mutant, *637ys,* in rice, accumulating small amounts of Chl_phy_ and a majority of Chls with unsaturated side chains. Map-based cloning and complementation experiments showed that the mutant phenotype of *637ys* was a result of the single nucleotide substitution of *OsLIL3* (*LOC_Os02g03330*) gene, which shows high similarity with *LIL3* in Arabidopsis. *OsLIL3* is a single copy gene and is mainly expressed in green tissues. Its encoded protein is located in the chloroplast. Therefore, we have successfully identified *LIL3* gene in rice through a map-based cloning approach.

In Arabidopsis, both *lil3:1* knock-down and *lil3:2* null mutants displayed an indistinguishable phenotype from the wild type, but the *lil3:1 lil3:2* double mutant exhibited yellowish green leaves and grew slower than the *lil3* single mutants and the wild type plants. 96% of Chl molecules in the *lil3:1 lil3:2* double mutant were conjugated with incompletely reduced side chains, while the α-tocopherol was barely detectable. These results suggested that LIL3:1 and LIL3:2 proteins share overlapping function and are required for complete biosynthesis of phytyl residues of Chl and tocopherols [18, 20]. In the *637ys* mutant, a single nucleotide substitution occurred in *OsLIL3*, leading to 10 bp-insertion in cDNA sequence and premature termination of translation. The mutant Oslil3 protein contains a total of 133 amino acids without transmembrane helices. After removal of 44 amino acids of chloroplast transit peptide, the rest of the premature protein was predicted to contain 78 of wild-type plus 11 of new amino acids (Additional file 8: Figure S4a). The *637ys* mutant displayed a yellow-green leaf phenotype with a very slow growth rate and arrested chloroplast development. Correspondingly, the *637ys* mutant accumulated about 10% of Chl_phy_ in leaves, and 11.8 and 9.1% of α-tocopherol in leaves and grains respectively, equivalent to those of the wild type (Additional file 1: Figure S1; Fig. 5c, e, f). These data suggested that the function of the mutant Oslil3 protein might not be completely abolished, thus the remaining 78 amino acids at N-terminal could still contribute to GGR reaction, or that GGR in *637ys* showed a low level of activity without LIL3 protein. Taken together, these data implied that LHC-like protein LIL3 plays a crucial role in phytyl biosynthesis of Chl and tocopherols.

LIL3 proteins directly interact with GGR and are required for stable accumulation of GGR in Arabidopsis [18]. Takahashi et al. [40] conjugated the transmembrane domain of LIL3 to GGR and expressed these chimeric proteins in the *lil3:1 lil3:2* double mutant lacking LIL3 protein. As a result, the transgenic plants restored phytol-synthesizing activity and partially rescued the deficiency of Chl_phy_, suggesting that Arabidopsis LIL3 contribute to GGR reaction by anchoring GGR to the membrane through LHC motif. In rice, the *chlp* mutant *lyl1* has been reported to accumulate Chl with unsaturated side chains, defect in tocopherols in leaves, and be sensitive to light intensity [14]. Till recently, OsLIL3 has been confirmed to interact with OsGGR as well [41]. In the present study, the *502ys* mutant was more comprehensively investigated to do a comparison analysis with *637ys*. *637ys* showed similar characteristics with *502ys*, including sensitivity to temperature and light intensity (Fig. 4; Additional file 12: Figure S8), tocopherol and tocotrienol compositions in both leaves and grains (Fig. 5; Additional file 13: Figure S9), and alteration in expression of 18 genes involved in photosynthesis and Chl synthesis (Fig. 11; Additional file 14: Fig. S10). Moreover, *OsLIL3* and *OsCHLP* genes displayed a consistent expression pattern (Fig. 10; Additional file 15: Figure S11). Together with the lack of substantial Chl_phy_ and tocopherols in *637ys*, these data suggested that OsLIL3 is of extreme importance to the function of OsGGR. Recently, Hey et al. [20] demonstrated Arabidopsis LIL3 also interact directly with protochlorophyllide oxidoreductase (POR), and LIL3-deficient plants exhibited substantial loss of POR, suggesting LIL3 had another function for the stability of POR. Collectively, the versatility of LIL3 in Chl biosynthesis revealed its important role in growth and development of plants.

Chl consists of two moieties, a chlorin ring and a phytyl chain. Its chlorin rings absorb light energy, while phytyl chains are required for integrating Chls into hydrophobic regions of thylakoid membranes in chloroplasts [5, 42]. Naturally, Chl_GG_ is instantaneously able to be detected in etioplasts of higher plants during de-etiolation [43]. Transgenic tobacco expressing antisense *chlP*-RNA showed decreased levels of Chl and tocopherol and accumulated substantial levels of Chl_GG_ in leaves, displaying a delayed growth rate [9]. Rice mutants M134 and M249 accumulated only 3.4 and 1.4% of Chl_phy_ under the low light of 45 μmol m^− 2^ s^− 1^, and died when seedlings were grown for about 2 weeks under natural sunlight. However, the mutant seedlings could survive at least 2 months under the low-light condition [30]. Similarly, the *lyl1–2* mutant preferentially accumulated Chl_GG_, grew very slowly, and died after it was transferred to natural sunlight [14]. In present study, the *637ys* mutant accumulated more Chls with unsaturated side chains than the *502ys* mutant in which GGR should be still active due to the single amino acid substitution (Gly to Ser) and, correspondingly, exhibited more severe phenotypes (Table 1; Figs. 1, 2, 12) [15]. Furthermore, the *637ys 502ys* double mutant (*lil3 chlp*) displayed much more severe phenotypes than the single mutants and exclusive accumulation of Chl_GG_. Consequently, it died at the three-leaf stage not only under natural sunlight (Figs. 12, 13), but also under the low-light condition in the growth chamber (Additional file 11: Figure S7). These data suggested that Chl_phy_ is critical to plant growth and development, and the complete replacement of phytyl side chain of chlorophyll by geranylgeranyl chain could be fatal to plant survival in rice.

## Conclusions

In this study, we identified the *OsLIL3* gene through a map-based cloning approach. Meanwhile, we demonstrated that OsLIL3 is of extreme importance to the function of OsGGR, and that the complete replacement of phytyl side chain of chlorophyll by geranylgeranyl chain could be fatal to plant survival in rice.

## Methods

### Plant materials

The plant materials used in this study were originally from our lab. The yellow-green mutants, *637ys* and *502ys*, were obtained from commonly used *japonica* cultivar Zhonghua 11 (ZH11) and Nipponbare (NP), respectively, via ethyl methanesulfonate (EMS) mutagenesis. F_2_ population was constructed for mapping by crossing *637ys* with normal green *indica* cultivar Gang 46B (G46B). The *637ys 502ys* double mutant was generated by crossing *637ys* and *502ys* and sequencing the F_2_ mutant plants, in which primer sets 5′-AAAGTGAAGTCCCAAGCG-3′ and 5′- CTAATCATGCTCCACCGA-3′, and 5′- ACATAATTCAGCACCACATG-3′ and 5′-ACACCCAGCCGTAGAAGT-3′ are used for detecting the mutation sites of *OsLIL3* in *637ys* and *OsCHLP* in *502ys*, respectively. Rice materials were planted during the normal rice growing season in an experimental field of Sichuan Agricultural University in Wenjiang District, Chengdu City, China [44]. For temperature treatments, seedlings were planted in a growth chamber with 12 h of light/12 h of dark at constant 23 °C (low temperature) or 30 °C (high temperature). For light intensity treatments, seedlings were cultivated in a growth chamber with 12 h of low light (80 μmol m^− 2^ s^− 1^)/12 h of dark or 12 h of high light (300 μmol m^− 2^ s^− 1^)/12 h of dark at a constant temperature.

### Analysis of pigments

Pigments were extracted from 0.2 g fresh leaves with 80% acetone followed by 48 h of incubation in dark at 4 °C. The contents of photosynthetic pigments including Chl and carotenoid (Caro) were determined with UV-1700 UV–visible spectrophotometer (Shi-madzu) at 663 nm, 646 nm and 470 nm, and were calculated by using the equations described by Lichtenthaler and Wellburn [45].

Chl used for HPLC analysis were extracted from fresh rice leaf tissue at the three-leaf stage with 100% acetone by centrifuging at 7197 g (Eppendorf 5430R; 7830 rpm) for 15 min. The supernatants filtered with 0.22 μm membrane were subjected to HPLC on a C18 column (Eclipse XDB-C18, 4.6 mm i.d. × 150 mm, 5 μm; Agilent). The mobile phase was solvent containing methanol, acetonitrile and acetone (1:3:1 v:v:v) at a flow-rate of 1.0 mL min^− 1^ at 40 °C [46]. Elution profiles were monitored at 660 nm.

### Transmission electron microscopy analysis

The fully expanded leaves of the wild-type ZH11 and the *637ys* mutant were harvested from seedlings grown in the field under natural planting season at the three-leaf stage. The second leaf of the *637ys 502ys* double mutant was harvested when the third leaf started to emerge from the sheath. The treatment on leaf sections and observation of chloroplast ultrastructure followed the method described by Wang et al. [38].

### Analysis of tocopherols and tocotrienols

Tocopherols in leaves were extracted from 0.1 g of fresh leaf tissue according to the method reported by Zhang et al. [47] with minor modification. 0.1 g of fresh leaf tissue was ground in liquid nitrogen, and then added with 600 μL of methanol: chloroform (2:1 v:v) followed by 1 h of incubation in dark. After added with 200 μL of chloroform and 360 μL of H_2_O, the samples were mixed well by vortex for 30 s, and centrifuged at 3000 rpm. The organic layer was collected and dried under N_2_ gas. The dry residue was dissolved in 300 μL of ethanol and filtered with 0.22 μm membrane for HPLC analysis.

Tocopherols and tocotrienols in grains were extracted according to the method reported by Panfili et al. [48] with minor modification. 2 g of brown rice powder was saponified in a 50 mL tube with well mixed 10 mL of ethanol, 5 mL of pyrogallol (60 g/L), 2 mL of sodium chloride (10 g/L), incubated in 70 °C water bath for 5 min, and then added with 1 mL of potassium hydroxide (500 g/L). The tubes were placed in a 70 °C water bath and mixed every 5–10 min during saponification. After alkaline digestion at 70 °C for 30 min, the tubes were cooled in an ice bath, and 10 mL of pre-cooled sodium chloride (10 g/L) was added. The suspension was then extracted more than three times with a 15 mL portion of n-hexane/ethyl acetate (4:1 v/v). The organic layer was collected and dried under N_2_ gas, and the dry residue was dissolved in 600 μL of ethanol and filtered with 0.22 μm membrane for HPLC analysis. 10 μg of δ-tocopherol standard, which does not exist in rice grains, was added to each sample prior to extraction as an internal standard [49].

The chromatographic separation of the compounds was achieved with HPLC from Agilent 1260. A C18 column (Eclipse XDB-C18, 4.6 mm i.d. × 150 mm, 5 μm; Agilent) was used. The mobile phase was methanol/H_2_O (95:1 V/V) at a flow rate of 1.5 mL/min at 30 °C [4, 14]. Fluorometric detection of all peaks was performed at an excitation wavelength of 290 nm and an emission wavelength of 330 nm. Injection volume was 20 μL. The tocopherols were quantified by using α-, γ-, and δ-tocopherol standards (Sigma).

### Marker development

The information of SSR markers was acquired from Gramene database (http://archive.gramene.org/markers/microsat/) according to the SSR linkage map constructed by McCouch et al. [50]. By BLAST searching in the National Center for Biotechnology Information database (http://www.ncbi.nlm.nih.gov/BLAST/), the sequence divergence including 15 to 100 bp insertion/deletion (InDel) between *japonica* cultivar NP and *indica* cultivar 9311 was used to design markers for fine mapping by using the Primer 5.0 software.

### Sequence analysis

The DNA and amino acid sequences of OsLIL3 and its homologues were acquired from GenBank (http://www.ncbi.nlm.nih.gov) by BLAST. The prediction of chloroplast transit peptide was carried out by TargetP and ChloroP (http://www.cbs.dtu.dk/services/TargetP/; http://www.cbs.dtu.dk/services/ChloroP/) [31, 32]. Multiple sequence alignment and phylogenetic analysis were conducted using DNAMAN version 6.0 (Lynnon Biosoft).

### Complementation analysis of the *637ys* mutant

For the complementation assay, the full-length cDNA sequence (753 bp) of *OsLIL3* (*LOC_Os02g03330*) was amplified from the cDNA of wild-type ZH11 by using the primer set 5′-GGGTCTAGAATGGCCATGGCGACCTCC-3′ and 5′-CTCTGCAGCTATTTCTTGGGCTGAGAAG-3′, containing an *Xba* I site and a *Pst* I site, respectively. After treating with enzymes *Xba* I and *Pst* I, the amplified fragment was inserted into the binary vector pCAMBIA2300 to produce construct PC2300-*OsLIL3* driven by the *Actin 1* promoter. The pC2300-*OsLIL3* plasmid was transformed into *637ys* mediated by *Agrobacterium tumefaciens* strain EHA105. The positive transgenic plants were confirmed by using the primer set 5′-AAGTTCCGGGACTCGAGG-3′ and 5′-GCGATCATAGGCGTCTCG-3′, which located on the *OsLIL3* gene and the pCAMBIA2300 vector, respectively.

### Subcellular localization of the OsLIL3 protein

The full-length cDNA sequence of *OsLIL3* was amplified from the cDNA of ZH11 by using the primer set 5′- GGGGAATTCATGGCCATGGCGACCTCC-3′and 5′-CTTCTAGAGTATTTCTTGGGCTGAGAAG-3′, harboring a *Bam*H I site at the 5′-end and an *Xba* I site at the 3′-end of the *OsLIL3* gene, respectively. After amplification, the fragment was introduced into the pC2300–35S-GFP vector. The generated construct pC2300–35S-*OsLIL3*-GFP was subsequently transformed into the wild-type protoplasts, with pC2300–35S-GFP as control, following the protocol described previously [51]. Then the transformed protoplast suspension was incubated overnight in dark, followed by observation under a laser-scanning confocal microscopy (Nikon A1).

### qRT-PCR analysis

Rice samples were harvested from 7 to 8 a.m. Total rice RNA from root, leaf, leaf sheath, young panicle and stem was extracted with a RNA isolater kit (Vazyme). The first-strand cDNA was reverse transcribed from total RNA (2 μg) by using a reverse transcription kit (Vazyme). The amplification was performed in a total volume of 10 μL with 0.1 μM of each primer and 1 × SYBR green PCR master mix (Vazyme) by using the CFX96 real-time PCR system (Bio-Rad). The reaction condition was as follow: 95 °C for 3 min, then 40 cycles of 95 °C for 10 s and 55 °C for 30 s. More than 3 plants were mixed for each sample. For each sample, three technical replicates on each of three biological replicates were carried out. The *Actin 1* gene was chosen as an internal control. The 2^-ΔΔCT^ method was used to calculate relative changes in gene expression. The Student’s *t* test was used for statistical analysis. All qRT-PCR primer sets were listed in Additional file 16: Table S5.

## Supplementary information

**Additional file 1: Figure S1.** Chlorophyll composition of the *637ys* mutant and its wild-type ZH11. **(a)** and **(b)** Elution profiles detected at 660 nm by using HPLC. **(c)** Absorption spectrum of peaks 1, 6 and 7 in acetone. **(d)** Absorption spectrum of peaks 2, 3, 4 and 5 in acetone. Peaks 2, 3, 4, and 5 represent Chl_phy_ *a*, Chl_THGG_ *a*, Chl_DHGG_ *a*, and Chl_GG_ *a*, respectively. Peaks 1, 6, and 7 represent Chl_phy_ *b*, Chl_DHGG_ *b*, and Chl_GG_ *b*, respectively. (PDF 420 kb)

**Additional file 2: Figure S2.** Phenotypic comparison of three-week-old seedlings grown in the growth chamber under low light (LL) or high light (HL) at constant temperature (23 °C or 30 °C). **(a1)**, **(a2)**, **(b1)**, and **(b2)** Phenotypes of ZH11 and *637ys* under 23 °C/LL, 30 °C/LL, 23 °C/HL, and 30 °C/HL, respectively. **(c1)**, **(c2)**, **(d1)**, and **(d2)** Phenotypes of NP and *502ys* under 23 °C/LL, 30 °C/LL, 23 °C/HL, and 30 °C/HL, respectively. (PDF 487 kb)

**Additional file 3: Table S1.** Pigment contents in leaves of the *637ys* and *502ys* mutants and their wild-type ZH11 and Nipponbare at different temperature and light-intensity treatments, in mg g fresh weight^− 1^ (PDF 253 kb)

**Additional file 4: Table S2.** Comparison of pigment contents in leaves of the *637ys* and *502ys* mutants and their wild-type ZH11 and Nipponbare between two different temperature treatments, in mg g fresh weight^− 1^ (PDF 421 kb)

**Additional file 5: Table S3.** Comparison of pigment contents in leaves of the *637ys* and *502ys* mutants and their wild-type ZH11 and Nipponbare between two different light-intensity treatments, in mg g fresh weight^− 1^ (PDF 421 kb)

**Additional file 6: Figure S3.** The peak area of tocotrienols in grains of ZH11 (WT) and *637ys*. α-T3, γ-T3 and δ-T3 represent α-tocotrienol, γ-tocotrienol and δ-tocotrienol, respectively. (PDF 440 kb)

**Additional file 7: Table S4.** Insertion/deletion (InDel) markers used for mapping of the *637ys* locus. (PDF 232 kb)

**Additional file 8: Figure S4.** Sequence alignment of OsLIL3 and its homologues. Identical residues are boxed in black, and similar residues (≥75% identical) are highlighted in gray. A black underline indicates the putative chloroplast signal peptides, a red arrow shows the mutant site of *637ys*, and green underlines represent the transmembrane helices predicted by HMMTOP. The blue box shows the LHC motif based on the sequence of LHC motif. The red box shows the 11 new amino acids in the mutant Oslil3 protein. GenBank accession numbers for the respective protein sequences are as Fig. 5. (PDF 482 kb)

**Additional file 9: Figure S5.** Predicted transmembrane domain of the OsLIL3 (LOC_Os02g03330) protein. (PDF 201 kb)

**Additional file 10: Figure S6.** Ultrastructure of mesophyll cells **(a)** and chloroplasts **(b)** in the *637ys 502ys* double mutant. Bars = 1 μm. (PDF 563 kb)

**Additional file 11: Figure S7.** Phenotypic comparison and Chl composition analysis of the wild-type ZH11 and Nipponbare (NP), the *637ys* mutant, the *502ys* mutant and the *637ys 502ys* double mutant (DM) at the three-leaf stage grown in a growth chamber under low light at constant 23 °C. **(a)** Phenotypic comparison. **(b)**, **(c)**, **(d)** and **(e)** The elution profiles of Chls in wild type, *637ys*, *502ys* and DM, respectively. Peaks 2, 3, 4, and 5 represent Chl_phy_ *a*, Chl_THGG_ *a*, Chl_DHGG_ *a*, and Chl_GG_ *a*, respectively. Peaks 1, 6 and 7 represent Chl_phy_ *b*, Chl_DHGG_ *b*, and Chl_GG_ *b*, respectively. The absorption spectra of elution profiles in acetone are the same as those in **Fig. S1c** and **d**. (PDF 546 kb)

**Additional file 12: Figure S8.** Total Chl contents in *502ys* and its wild-type (WT) grown under low light (LL) or high light (HL) at constant temperature (23 °C or 30 °C), in mg g fresh weight^− 1^. Data are shown as mean ± SD. Error bars represent standard deviations of three independent biological replicates. Asterisks indicate statistically significant differences compared with the wild-type at *P* < 0.01. (PDF 560 kb)

**Additional file 13: Figure S9** Analysis of vitamin E in leaves and grains of *502ys*. Elution profiles of the tocopherol standards **(a)**, tocopherols in leaves of wild-type Nipponbare **(b)** and *502ys* **(c)**, tocopherols and tocotrienols in grains of Nipponbare **(d)** and *502ys* **(e)** were detected by fluorescence with excitation at 290 nm and emission at 330 nm. **(f)** Tocopherol contents in leaves and grains of Nipponbare and *502ys* were quantified by using tocopherol standards. **(g)** The peak area of tocotrienols in grains of Nipponbare (WT) and *502ys*. α-T, α-tocopherol; γ-T, γ-tocopherol. The tocopherol standards were prepared as described in Fig. 5. α-T3, γ-T3 and δ-T3 represent α-tocotrienol, γ-tocotrienol and δ-tocotrienol, respectively. Peaks 1 and 2 represent α-tocopherol and γ-tocopherol; Peak 3 is δ-tocopherol which does not exist in rice and was used as control. Peaks 4, 5 and 6 represent α-tocotrienol, γ-tocotrienol and δ-tocotrienol, respectively. Peak 7 might be the isomer of γ-tocopherol. Error bars represent standard errors of three independent biological replicates. Asterisks indicate statistically significant differences compared with the wild-type at *P* < 0.01. (PDF 758 kb)

**Additional file 14: Figure S10.** Expression analysis of genes involved in photosynthesis and Chl biosynthesis in *502ys*. *Actin 1* was amplified as an internal reference. The expression level of each gene in wild types was set to 1.0, and those in *637ys* and *502ys* mutants were calculated accordingly. Error bars represent standard errors of three independent biological replicates. The asterisk indicates statistically significant differences compared with the wild-type at *P* < 0.05. (PDF 591 kb)

**Additional file 15: Figure S11.** Expression patterns of *OsCHLP* gene. The expression levels were determined by real-time PCR in root (R), and leaf (L), leaf sheath (LS), panicle (P), and stem (S) of wild type grown in a paddy field. The levels of transcripts were normalized to rice *Actin 1* gene as an internal control. Error bars represent standard errors of three independent biological replicates. (PDF 569 kb)

**Additional file 16: Table S5.** Primers used in RT-PCR. (PDF 277 kb)

## Data Availability

All data generated or analyzed during this study are included in this published article and its supplementary information files.

## Abbreviations

Chl: Chlorophyll
Chl_DHGG_: Dihydrogeranylgeranyl chlorophyll
Chl_GG_: geranylgeranyl chlorophyll
Chl_phy_: phytyl chlorophyll
Chl_THGG_: tetrahydrogeranylgeranyl chlorophyll
GGPP: Geranylgeranyl diphosphate
GGR: Geranylgeranyl reductase
LHC: Light-harvesting complex
LIL: Light-harvesting like protein
Phytyl-PP: Phytyl pyrophosphate

## References

[1] Fromme P, Melkozernov A, Jordan P, Krauss N (2003). Structure and function of photosystem I: interaction with its soluble electron carriers and external antenna systems. FEBS Lett.

[2] Wu ZM, Zhang X, He B, Diao LP, Sheng SL, Wang JL (2007). A chlorophyll-deficient rice mutant with impaired chlorophyllide esterification in chlorophyll biosynthesis. Plant Physiol.

[3] Cahoon EB, Hall SE, Ripp KG, Ganzke TS, Hitz WD, Coughlan SJ (2003). Metabolic redesign of vitamin E biosynthesis in plants for tocotrienol production and increased antioxidant content. Nat Biotechnol.

[4] Yang WY, Cahoon RE, Hunter SC, Zhang CY, Han JX, Borgschulte T (2011). Vitamin E biosynthesis: functional characterization of the monocot homogentisate geranylgeranyl transferase. Plant J.

[5] Keller Y, Bouvier F, d'Harlingue A, Camara B (1998). Metabolic compartmentation of plastid prenyllipid biosynthesis. Eur J Biochem.

[6] Ischebeck T, Zbierzak AM, Kanwischer M, Dormann P (2006). A salvage pathway for phytol metabolism in *Arabidopsis*. J Biol Chem.

[7] Valentin HE, Lincoln K, Moshiri F, Jensen PK, Qi QG, Venkatesh TV (2006). The *Arabidopsis vitamin E pathway gene5-1* mutant reveals a critical role for phytol kinase in seed tocopherol biosynthesis. Plant Cell.

[8] vom Dorp K, Holzl G, Plohmann C, Eisenhut M, Abraham M, Weber APM (2015). Remobilization of phytol from chlorophyll degradation is essential for tocopherol synthesis and growth of Arabidopsis. Plant Cell.

[9] Tanaka R, Oster U, Kruse E, Rüdiger W, Grimm B (1999). Reduced activity of geranylgeranyl reductase leads to loss of chlorophyll and tocopherol and to partially geranylgeranylated chlorophyll in transgenic tobacco plants expressing antisense RNA for geranylgeranyl reductase. Plant Physiol.

[10] Bollivar DW, Wang SJ, Allen JP, Bauer CE (1994). Molecular genetic analysis of terminal steps in bacteriochlorophyll a biosynthesis: characterization of a *Rhodobacter capsulatus* strain that synthesizes geranylgeraniol-esterified bacteriochlorophyll *a*. Biochemistry.

[11] Addlesee HA, Gibson LCD, Jensen PE, Hunter CN (1996). Cloning, sequencing and functional assignment of the chlorophyll biosynthesis gene, *chZP*, of *Synechocystis* sp. PCC6803. FEBS Lett.

[12] Addlesee HA, Hunter CN (1999). Physical mapping and functional assignment of the geranylgeranyl-bacteriochlorophyll reductase gene, *bchP*, of *Rhodobacter sphaeroides*. J Bacteriol.

[13] Chew AGM, Frigaard NU, Bryant DA (2008). Identification of the *bchP* gene, encoding geranylgeranyl reductase in *Chlorobaculum tepidum*. J Bacteriol.

[14] Zhou Y, Gong ZY, Yang ZF, Yuan Y, Zhu JY, Wang M (2013). Mutation of the *Light-induced Yellow Leaf 1* gene, which encodes a geranylgeranyl reductase, affects chlorophyll biosynthesis and light sensitivity in rice. PLoS One.

[15] Wang PY, Li CM, Wang Y, Huang R, Sun CH, Xu ZJ (2014). Identification of a geranylgeranyl reductase gene for chlorophyll synthesis in rice. Springer Plus.

[16] Shpilyov AV, Zinchenko VV, Shestakov SV, Grimm B, Lokstein H (2005). Inactivation of the geranylgeranyl reductase (ChlP) gene in the cyanobacterium Synechocystis sp. PCC 6803. Biochim Biophys Acta.

[17] Klimmek F, Sjödin A, Noutsos C, Leister D, Jansson S (2006). Abundantly and rarely expressed *Lhc* protein genes exhibit distinct regulation patterns in plants. Plant Physiol.

[18] Tanaka R, Rothbart M, Oka S, Takabayashi A, Takahashi K, Shibata M (2010). LIL3, a light-harvesting-like protein, plays an essential role in chlorophyll and tocopherol biosynthesis. Proc Natl Acad Sci U S A.

[19] Lohscheider JN, Rojas-Stutz MC, Rothbart M, Andersson U, Funck D, Mendgen K (2015). Altered levels of LIL3 isoforms in *Arabidopsis* lead to disturbed pigment-protein assembly and chlorophyll synthesis, chlorotic phenotype and impaired photosynthetic performance. Plant Cell Environ.

[20] Hey D, Rothbart M, Herbst J, Wang P, Müller J, Wittmann D (2017). LIL3, a light-harvesting complex protein, links terpenoid and tetrapyrrole biosynthesis in *Arabidopsis thaliana*. Plant Physiol.

[21] Hey D, Grimm B (2018). ONE-HELIX PROTEIN2 (OHP2) is required for the stability of OHP1 and assembly factor HCF244 and is functionally linked to PSII biogenesis. Plant Physiol.

[22] Myouga F, Takahashi K, Tanaka R, Nagata N, Kiss AZ, Funk C (2018). Stable accumulation of photosystem II requires ONE-HELIX PROTEIN1 (OHP1) of the light harvesting-like family. Plant Physiol.

[23] Heddad M, Adamska I (2000). Light stress-regulated two-helix proteins in *Arabidopsis thaliana* related to the chlorophyll *a*/*b*-binding gene family. Proc Natl Acad Sci U S A.

[24] Li XP, Björkman O, Shih C, Grossman AR, Rosenquist M, Jansson S (2000). A pigment-binding protein essential for regulation of photosynthetic light harvesting. Nature.

[25] Niyogi KK, Li XP, Rosenberg V, Jung HS (2005). Is PsbS the site of non-photochemical quenching in photosynthesis?. J Exp Bot.

[26] Tzvetkova-Chevolleau T, Franck F, Alawady AE, Dall'Osto L, Carriere F, Bassi R (2007). The light stress-induced protein ELIP2 is a regulator of chlorophyll synthesis in *Arabidopsis thaliana*. Plant J.

[27] Sobotka R, Tichy M, Wilde A, Hunter CN (2011). Functional assignments for the carboxyl-terminal domains of the ferrochelatase from *Synechocystis* PCC 6803: the CAB domain plays a regulatory role, and region II is essential for catalysis. Plant Physiol.

[28] Zhao L, Cheng DM, Huang XH, Chen M, Dall'Osto L, Xing JL (2017). A light harvesting complex-like protein in maintenance of photosynthetic components in *Chlamydomonas*. Plant Physiol.

[29] Reisinger V, Plöscher M, Eichacker LA (2008). Lil3 assembles as chlorophyll-binding protein complex during deetiolation. FEBS Lett.

[30] Shibata M, Mikota T, Yoshimura A, Iwata N, Tsuyama M, Kobayashi Y (2004). Chlorophyll formation and photosynthetic activity in rice mutants with alterations in hydrogenation of the chlorophyll alcohol side chain. Plant science : an international journal of experimental plant biology Plant Sci.

[31] Emanuelsson O, Nielsen H, von Heijne G (1999). ChloroP, a neural network-based method for predictingchloroplast transit peptides and their cleavage sites. Protein Sci.

[32] Emanuelsson O, Brunak S, von Heijne G, Nielsen H (2007). Locating proteins in the cell using TargetP, SignalP and related tools. Nat Protoc.

[33] Krogh A, Larsson B, von Heijne G, Sonnhammer ELL (2001). Predicting transmembrane protein topology with a hidden Markov model: application to complete genomes. J Mol Biol.

[34] Tusnády GE, Simon I (2001). The HMMTOP transmembrane topology prediction server. Bioinformatics.

[35] Su N, Hu ML, Wu DX, Wu FQ, Fei GL, Lan Y (2012). Disruption of a rice pentatricopeptide repeat protein causes a seedling-specific albino phenotype and its utilization to enhance seed purity in hybrid rice production. Plant Physiol.

[36] Inagaki N, Kinoshita K, Kagawa T, Tanaka A, Ueno O, Shimada H (2015). Phytochrome B mediates the regulation of chlorophyll biosynthesis through transcriptional regulation of *ChlH* and *GUN4* in Rice seedlings. PLoS One.

[37] Lee S, Kim JH, Yoo ES, Lee CH, Hirochika H, An G (2005). Differential regulation of *chlorophyll a oxygenase* genes in rice. Plant Mol Biol.

[38] Wang PR, Gao JX, Wan CM, Zhang FT, Xu ZJ, Huang XQ (2010). Divinyl chlorophyll (ide) *a* can be converted to monovinyl chlorophyll (ide) *a* by a divinyl reductase in rice. Plant Physiol.

[39] Sakuraba Y, Rahman ML, Cho SH, Kim YS, Koh HJ, Yoo SC (2013). The rice *faded green leaf* locus encodes protochlorophyllide oxidoreductase B and is essential for chlorophyll synthesis under high light conditions. Plant J.

[40] Takahashi K, Takabayashi A, Tanaka A, Tanaka R (2014). Functional analysis of light-harvesting-like protein 3 (LIL3) and its light-harvesting chlorophyll-binding motif in *Arabidopsis*. J Biol Chem.

[41] Zhou F, Wang CY, Gutensohn M, Jiang L, Zhang P, Zhang DB (2017). A recruiting protein of geranylgeranyl diphosphate synthase controls metabolic flux toward chlorophyll biosynthesis in rice. Proc Natl Acad Sci U S A.

[42] Tanaka R, Tanaka A (2007). Tetrapyrrole biosynthesis in higher plants. Annu Rev Plant Biol.

[43] Liljenberg C (1974). Characterization and properties of a protochlorophyllide ester in leaves of dark grown barley with geranylgeraniol as esterifying alcohol. Physiol Plant.

[44] Wang Y, Zhong P, Zhang XY, Liu JQ, Zhang CY, Yang XR (2019). *GRA78* encoding a putative S-sulfocysteine synthase is involved in chloroplast development at the early seedling stage of rice. Plant Sci.

[45] Lichtenthaler HK, Wellburn AR (1983). Determination of total carotenoids and chlorophylls *a* and *b* of leaf extracts in different solvents. Biochem Soc T.

[46] Nakanishi H, Nozue H, Suzuki K, Kaneko Y, Taguchi G, Hayashida N (2005). Characterization of the *Arabidopsis thaliana* mutant *pcb2* which accumulates divinyl chlorophylls. Plant Cell Physiol.

[47] Zhang W, Liu TQ, Ren GD, Hortensteiner S, Zhou YM, Cahoon EB (2014). Chlorophyll degradation: the tocopherol biosynthesis-related phytol hydrolase in Arabidopsis seeds is still missing. Plant Physiol.

[48] Panfili G, Fratianni A, Irano M (2003). Normal phase high-performance liquid chromatography method for the determination of tocopherols and tocotrienols in cereals. J Agr Food Chem.

[49] Heinemann RJB, Xu Z, Godber JS (2008). Lanfer-Marquez U. tocopherols, Tocotrienols, and γ-Oryzanol contents in japonica and Indica subspecies of rice (Oryza sativa L.). Cereal Chem.

[50] McCouch SR, Teytelman L, Xu YB, Lobos KB, Clare K, Walton M (2002). Development and mapping of 2240 new SSR markers for rice (*Oryza sativa* L.). DNA Res.

[51] Zhang Y, Su J, Duan S, Ao Y, Dai J, Liu J (2011). A highly efficient rice green tissue protoplast system for transient gene expression and studying light/chloroplast-related processes. Plant Methods.

