# Supplementary material for: A lil3 chlp double mutant with exclusive accumulation of geranylgeranyl chlorophyll displays a lethal phenotype in rice
Source: BMC Plant Biol. 2019 Oct 29;19:456. doi: 10.1186/s12870-019-2028-z (PMC6819399; doi:10.1186/s12870-019-2028-z)
Supplement: Supplementary file 1 — Additional file 1: Figure S1. Chlorophyll composition of the 637ys mutant and its wild-type ZH11. (a) and (b) Elution profiles detected at 660 nm by using HPLC. (c) Absorption spectrum of peaks 1, 6 and 7 in acetone. (d) Absorption spectrum of peaks 2, 3, 4 and 5 in acetone. Peaks 2, 3, 4, and 5 represent Chlphy a, ChlTHGG a, ChlDHGG a, and ChlGG a, respectively. Peaks 1, 6, and 7 represent Chlphy b, ChlDHGG b, and ChlGG b, respectively. (PDF 420 kb) [file 12870_2019_2028_MOESM1_ESM.pdf]

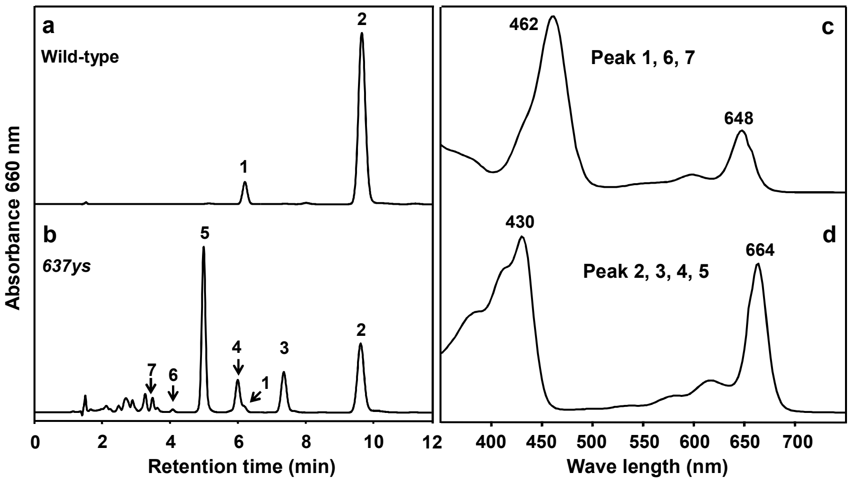

Additional file 1: Figure S1. Chlorophyll composition of the *637ys* mutant and its wild-type ZH11. **(a)** and **(b)** Elution profiles detected at 660 nm by using HPLC. **(c)** Absorption spectrum of peaks 1, 6 and 7 in acetone. **(d)** Absorption spectrum of peaks 2, 3, 4 and 5 in acetone. Peaks 2, 3, 4, and 5 represent Chl_{phy} *a*, Chl_{THGG} *a*, Chl_{DHGG} *a*, and Chl_{GG} *a*, respectively. Peaks 1, 6 and 7 represent Chl_{phy} *b*, Chl_{DHGG} *b*, and Chl_{GG} *b*, respectively.
