# Supplementary material for: A lil3 chlp double mutant with exclusive accumulation of geranylgeranyl chlorophyll displays a lethal phenotype in rice
Source: BMC Plant Biol. 2019 Oct 29;19:456. doi: 10.1186/s12870-019-2028-z (PMC6819399; doi:10.1186/s12870-019-2028-z)
Supplement: Supplementary file 2 — Additional file 2: Figure S2. Phenotypic comparison of three-week-old seedlings grown in the growth chamber under low light (LL) or high light (HL) at constant temperature (23 °C or 30 °C). (a1), (a2), (b1), and (b2) Phenotypes of ZH11 and 637ys under 23 °C/LL, 30 °C/LL, 23 °C/HL, and 30 °C/HL, respectively. (c1), (c2), (d1), and (d2) Phenotypes of NP and 502ys under 23 °C/LL, 30 °C/LL, 23 °C/HL, and 30 °C/HL, respectively. (PDF 487 kb) [file 12870_2019_2028_MOESM2_ESM.pdf]

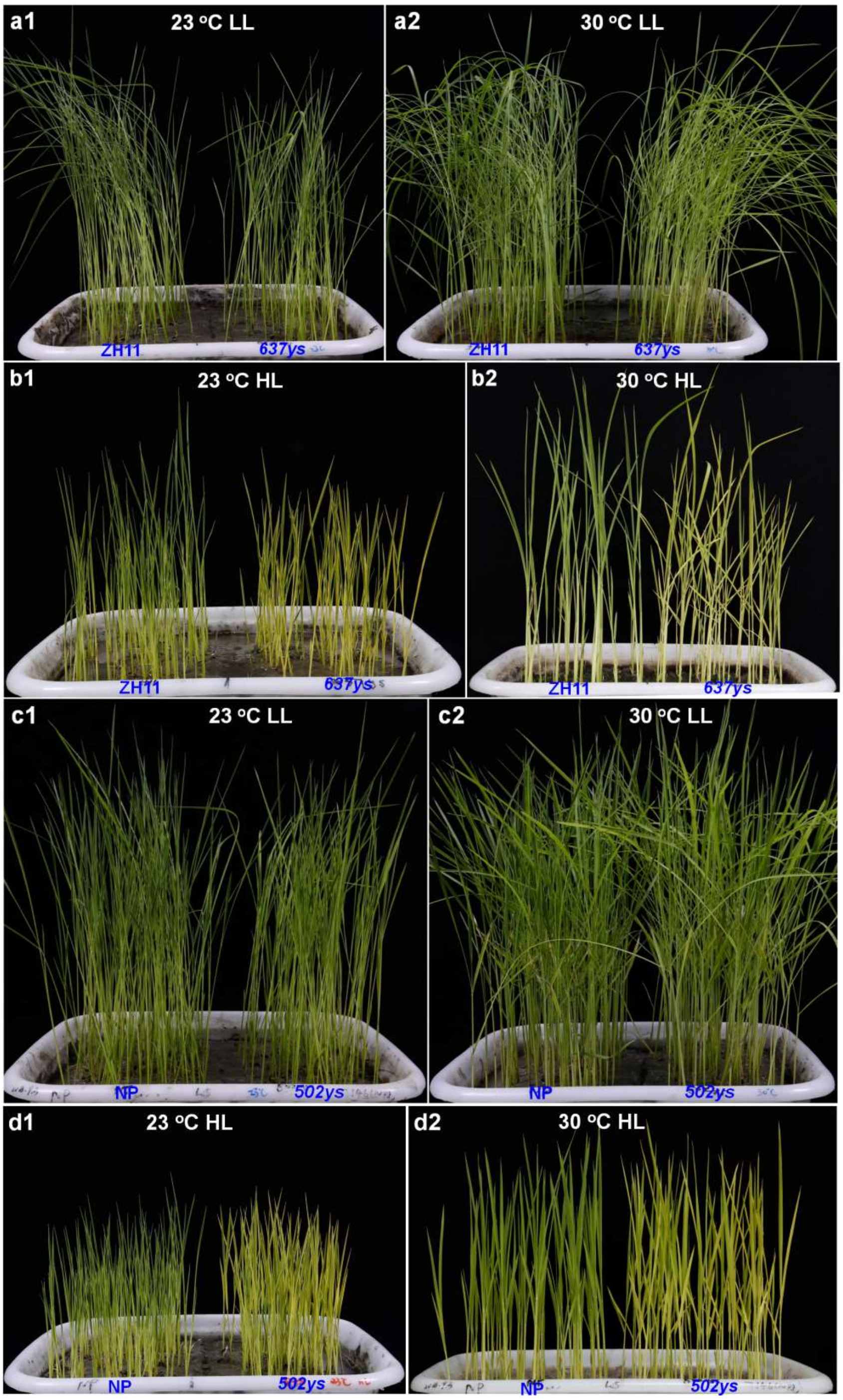

Additional file 2: Figure S2. Phenotypic comparison of three-week-old seedlings grown in the growth chamber under low light (LL) or high light (HL) at constant temperature (23 °C or 30 °C). **(a1)**, **(a2)**, **(b1)**, and **(b2)** Phenotypes of ZH11 and *637ys* under 23 °C/LL, 30 °C/LL, 23 °C/HL, and 30 °C/HL, respectively. **(c1)**, **(c2)**, **(d1)**, and **(d2)** Phenotypes of NP and *502ys* under 23 °C/LL, 30 °C/LL, 23 °C/HL, and 30 °C/HL, respectively.
