# Supplementary material for: A lil3 chlp double mutant with exclusive accumulation of geranylgeranyl chlorophyll displays a lethal phenotype in rice
Source: BMC Plant Biol. 2019 Oct 29;19:456. doi: 10.1186/s12870-019-2028-z (PMC6819399; doi:10.1186/s12870-019-2028-z)
Supplement: Supplementary file 3 — Additional file 3: Table S1. Pigment contents in leaves of the 637ys and 502ys mutants and their wild-type ZH11 and Nipponbare at different temperature and light-intensity treatments, in mg g fresh weight− 1 (PDF 253 kb) [file 12870_2019_2028_MOESM3_ESM.pdf]

Additional file 3: Table S1. Pigment contents in leaves of the *637ys* and *502ys* mutants and their wild-type ZH11 and Nipponbare at different temperature and light-intensity treatments, in mg g fresh weight^{-1}

Treatment Code	Treatment Condition	Material	Chl (mg/g)	Chl *a* (mg/g)	Chl *b* (mg/g)	Carotenoid (mg/g)
a1	23°C/LL	ZH11 (CK)	2.31±0.23	1.89±0.19	0.41±0.04	0.55±0.05
		637ys	1.49±0.33	1.19±0.27	0.29±0.06	0.40±0.08
		Compared with CK	-35.5%**	-37.0%**	-29.3%**	-27.3%**
a2	30°C/LL	ZH11 (CK)	2.14±0.11	1.73±0.03	0.41±0.02	0.54±0.03
		637ys	1.52±0.10	1.22±0.08	0.30±0.02	0.43±0.03
		Compared with CK	-29.0%**	-29.5%**	-26.8%**	-20.4%**
b1	23°C/HL	ZH11 (CK)	1.78±0.22	1.50±0.08	0.28±0.04	0.49±0.01
		637ys	0.84±0.04	0.70±0.01	0.14±0.01	0.40±0.02
		Compared with CK	-52.8%**	-53.3%**	-50.0%**	-18.4%**
b2	30°C/HL	ZH11 (CK)	1.93±0.14	1.58±0.11	0.35±0.03	0.50±0.03
		637ys	0.80±0.07	0.65±0.06	0.15±0.01	0.36±0.04
		Compared with CK	-58.5%**	-58.9%**	-57.1%**	-28.0%**
c1	23°C/LL	Nipponbare(CK)	2.10±0.10	1.73±0.08	0.37±0.01	0.51±0.03
		502ys	1.54±0.13	1.24±0.10	0.30±0.02	0.42±0.03
		Compared with CK	-26.7%**	-28.3%**	-18.9%**	-17.6%**
c2	30°C/LL	Nipponbare (CK)	1.86±0.34	1.51±0.27	0.36±0.07	0.48±0.09
		502ys	1.62±0.14	1.31±0.12	0.32±0.02	0.45±0.03
		Compared with CK	-12.9%	-13.2%	-11.1%	-6.3%
d1	23°C/HL	Nipponbare (CK)	1.51±0.21	1.27±0.01	0.23±0.04	0.37±0.05
		502ys	0.67±0.04	0.57±0.01	0.10±0.01	0.33±0.02
		Compared with CK	-55.6%**	-55.1%**	-56.5%**	-10.8%
d2	30°C/HL	Nipponbare (CK)	1.65±0.10	1.44±0.02	0.31±0.02	0.43±0.02
		502ys	0.74±0.15	0.61±0.12	0.13±0.03	0.28±0.03
		Compared with CK	-55.2%**	-57.6%**	-58.1%**	-34.9%**

** Significantly different at $P = 0.01$
