# Supplementary material for: A lil3 chlp double mutant with exclusive accumulation of geranylgeranyl chlorophyll displays a lethal phenotype in rice
Source: BMC Plant Biol. 2019 Oct 29;19:456. doi: 10.1186/s12870-019-2028-z (PMC6819399; doi:10.1186/s12870-019-2028-z)
Supplement: Supplementary file 4 — Additional file 4: Table S2. Comparison of pigment contents in leaves of the 637ys and 502ys mutants and their wild-type ZH11 and Nipponbare between two different temperature treatments, in mg g fresh weight− 1 (PDF 421 kb) [file 12870_2019_2028_MOESM4_ESM.pdf]

Additional file 4: Table S2. Comparison of pigment contents in leaves of the *637ys* and *502ys* mutants and their wild-type ZH11 and Nipponbare between two different temperature treatments, in mg g fresh weight^{-1}

Material	Treatment		Chl (mg/g)	Chl *a* (mg/g)	Chl *b* (mg/g)	Carotenoid (mg/g)
	Code	Condition				
ZH11 (CK)	a1	23°C/LL	2.31±0.23^a	1.89±0.19^a	0.41±0.04^a	0.55±0.05^a
	a2	30°C/LL	2.14±0.11^a	1.73±0.03^a	0.41±0.02^a	0.54±0.03^a
637ys	a1	23°C/LL	1.49±0.33^a	1.19±0.27^a	0.29±0.06^a	0.40±0.08^a
	a2	30°C/LL	1.52±0.10^a	1.22±0.08^a	0.30±0.02^a	0.43±0.03^a
ZH11 (CK)	b1	23°C/HL	1.78±0.22^a	1.50±0.08^a	0.28±0.04	0.49±0.01^a
	b2	30°C/HL	1.93±0.14^a	1.58±0.11^a	0.35±0.03^a	0.50±0.03^a
637ys	b1	23°C/HL	0.84±0.05^a	0.70±0.01^a	0.14±0.01^a	0.40±0.02^a
	b2	30°C/HL	0.80±0.07^a	0.65±0.06^a	0.15±0.01^a	0.36±0.04^a
Nipponbare (CK)	c1	23°C/LL	2.10±0.10^a	1.73±0.08^a	0.37±0.01^a	0.51±0.03^a
	c2	30°C/LL	1.86±0.34^a	1.51±0.27^a	0.36±0.07^a	0.48±0.09^a
502ys	c1	23°C/LL	1.54±0.13^a	1.24±0.10^a	0.30±0.02^a	0.42±0.03^a
	c2	30°C/LL	1.62±0.14^a	1.31±0.12^a	0.32±0.02^a	0.45±0.03^a
Nipponbare (CK)	d1	23°C/HL	1.51±0.21^a	1.27±0.01^a	0.23±0.04^a	0.37±0.05^a
	d2	30°C/HL	1.65±0.10^a	1.44±0.02^b	0.31±0.02^a	0.43±0.02^a
502ys	d1	23°C/HL	0.67±0.04^a	0.57±0.01^a	0.10±0.01^a	0.33±0.02^a
	d2	30°C/HL	0.74±0.15^a	0.61±0.12^a	0.13±0.03^a	0.28±0.03^a

^{a}, ^{b} No significant difference at P = 0.01 between 23°C and 30°C treatments with the same letter in the same experimental group.
