# Supplementary material for: A lil3 chlp double mutant with exclusive accumulation of geranylgeranyl chlorophyll displays a lethal phenotype in rice
Source: BMC Plant Biol. 2019 Oct 29;19:456. doi: 10.1186/s12870-019-2028-z (PMC6819399; doi:10.1186/s12870-019-2028-z)
Supplement: Supplementary file 5 — Additional file 5: Table S3. Comparison of pigment contents in leaves of the 637ys and 502ys mutants and their wild-type ZH11 and Nipponbare between two different light-intensity treatments, in mg g fresh weight− 1 (PDF 421 kb) [file 12870_2019_2028_MOESM5_ESM.pdf]

Additional file 5: Table S3. Comparison of pigment contents in leaves of the *637ys* and *502ys* mutants and their wild-type ZH11 and Nipponbare between two different light-intensity treatments, in mg g fresh weight^{-1}

Material	Treatment		Chl (mg/g)	Chl *a* (mg/g)	Chl *b* (mg/g)	Carotenoid (mg/g)
	Code	Condition				
ZH11 (CK)	a1	23°C/LL	2.31±0.23[a]	1.89±0.19[a]	0.41±0.04[a]	0.55±0.05[a]
	b1	23°C/HL	1.78±0.22[a]	1.50±0.08[b]	0.28±0.04[a]	0.49±0.01[a]
637ys	a1	23°C/LL	1.49±0.33[a]	1.19±0.27[a]	0.29±0.06[a]	0.40±0.08[a]
	b1	23°C/HL	0.84±0.04[b]	0.70±0.01[b]	0.14±0.01[b]	0.40±0.02[a]
ZH11 (CK)	a2	30°C/LL	2.14±0.11[a]	1.73±0.03[a]	0.41±0.02[a]	0.54±0.03[a]
	b2	30°C/HL	1.93±0.14[a]	1.58±0.11[a]	0.35±0.03[a]	0.50±0.03[a]
637ys	a2	30°C/LL	1.52±0.10 [a]	1.22±0.08[a]	0.30±0.02[a]	0.43±0.03[a]
	b2	30°C/HL	0.80±0.07[b]	0.65±0.06[b]	0.15±0.01[b]	0.36±0.04[a]
Nipponbare (CK)	c1	23°C/LL	2.10±0.10[a]	1.73±0.08[a]	0.37±0.01[a]	0.51±0.03[a]
	d1	23°C/HL	1.51±0.21[b]	1.27±0.01[b]	0.23±0.04[b]	0.37±0.05[b]
502ys	c1	23°C/LL	1.54±0.13 [a]	1.24±0.10[a]	0.30±0.02[a]	0.42±0.03[a]
	d1	23°C/HL	0.67±0.01[b]	0.57±0.01[b]	0.10±0.01[b]	0.33±0.02[b]
Nipponbare (CK)	c2	30°C/LL	1.86±0.34 [a]	1.51±0.27[a]	0.36±0.07[a]	0.48±0.09[a]
	d2	30°C/HL	1.65±0.10[a]	1.44±0.02[a]	0.31±0.02[a]	0.43±0.02[a]
502ys	c2	30°C/LL	1.62±0.14[a]	1.31±0.12[a]	0.32±0.02[a]	0.45±0.03[a]
	d2	30°C/HL	0.74±0.15[b]	0.61±0.12[b]	0.13±0.03[b]	0.28±0.03[b]

[a], [b] No significant difference at $P = 0.01$ between low-light (LL) and high-light (HL) treatments with the same letter in the same experimental group.
