# Supplementary material for: A lil3 chlp double mutant with exclusive accumulation of geranylgeranyl chlorophyll displays a lethal phenotype in rice
Source: BMC Plant Biol. 2019 Oct 29;19:456. doi: 10.1186/s12870-019-2028-z (PMC6819399; doi:10.1186/s12870-019-2028-z)
Supplement: Supplementary file 6 — Additional file 6: Figure S3. The peak area of tocotrienols in grains of ZH11 (WT) and 637ys. α-T3, γ-T3 and δ-T3 represent α-tocotrienol, γ-tocotrienol and δ-tocotrienol, respectively. (PDF 440 kb) [file 12870_2019_2028_MOESM6_ESM.pdf]

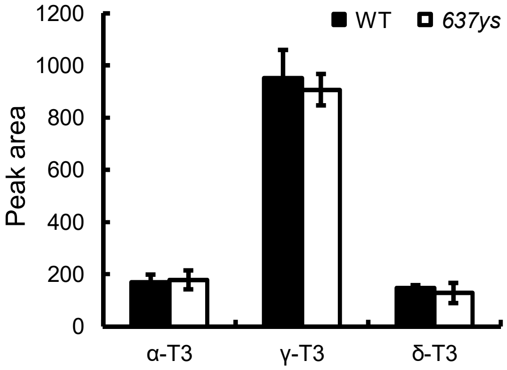

Additional file 6: Figure S3. The peak area of tocotrienols in grains of ZH11 (WT) and *637ys*. α-T3, γ-T3 and δ-T3 represent α-tocotrienol, γ-tocotrienol and δ-tocotrienol, respectively.
