# Supplementary material for: A lil3 chlp double mutant with exclusive accumulation of geranylgeranyl chlorophyll displays a lethal phenotype in rice
Source: BMC Plant Biol. 2019 Oct 29;19:456. doi: 10.1186/s12870-019-2028-z (PMC6819399; doi:10.1186/s12870-019-2028-z)
Supplement: Supplementary file 7 — Additional file 7: Table S4. Insertion/deletion (InDel) markers used for mapping of the 637ys locus. (PDF 232 kb) [file 12870_2019_2028_MOESM7_ESM.pdf]

Additional file 7: Table S4 Insertion/deletion (InDel) makers used for mapping of the *637ys* locus

Maker	Forward primer (5′–3′)	Reverse primer (5′–3′)
L1	AGACAGAACAGCGGTCAA	GTTAGTGCTTCGGAGTGG
L2	CCTTGGAGGTAAAGCATC	GGGACGGAGGGAGTAGTT
L3	TAGCCTGCCAGGATGGAC	CAAGATGCTTCGTAAATC
