# Supplementary material for: A lil3 chlp double mutant with exclusive accumulation of geranylgeranyl chlorophyll displays a lethal phenotype in rice
Source: BMC Plant Biol. 2019 Oct 29;19:456. doi: 10.1186/s12870-019-2028-z (PMC6819399; doi:10.1186/s12870-019-2028-z)
Supplement: Supplementary file 8 — Additional file 8: Figure S4. Sequence alignment of OsLIL3 and its homologues. Identical residues are boxed in black, and similar residues (≥75% identical) are highlighted in gray. A black underline indicates the putative chloroplast signal peptides, a red arrow shows the mutant site of 637ys, and green underlines represent the transmembrane helices predicted by HMMTOP. The blue box shows the LHC motif based on the sequence of LHC motif. The red box shows the 11 new amino acids in the mutant Oslil3 protein. GenBank accession numbers for the respective protein sequences are as Fig. 5. (PDF 482 kb) [file 12870_2019_2028_MOESM8_ESM.pdf]

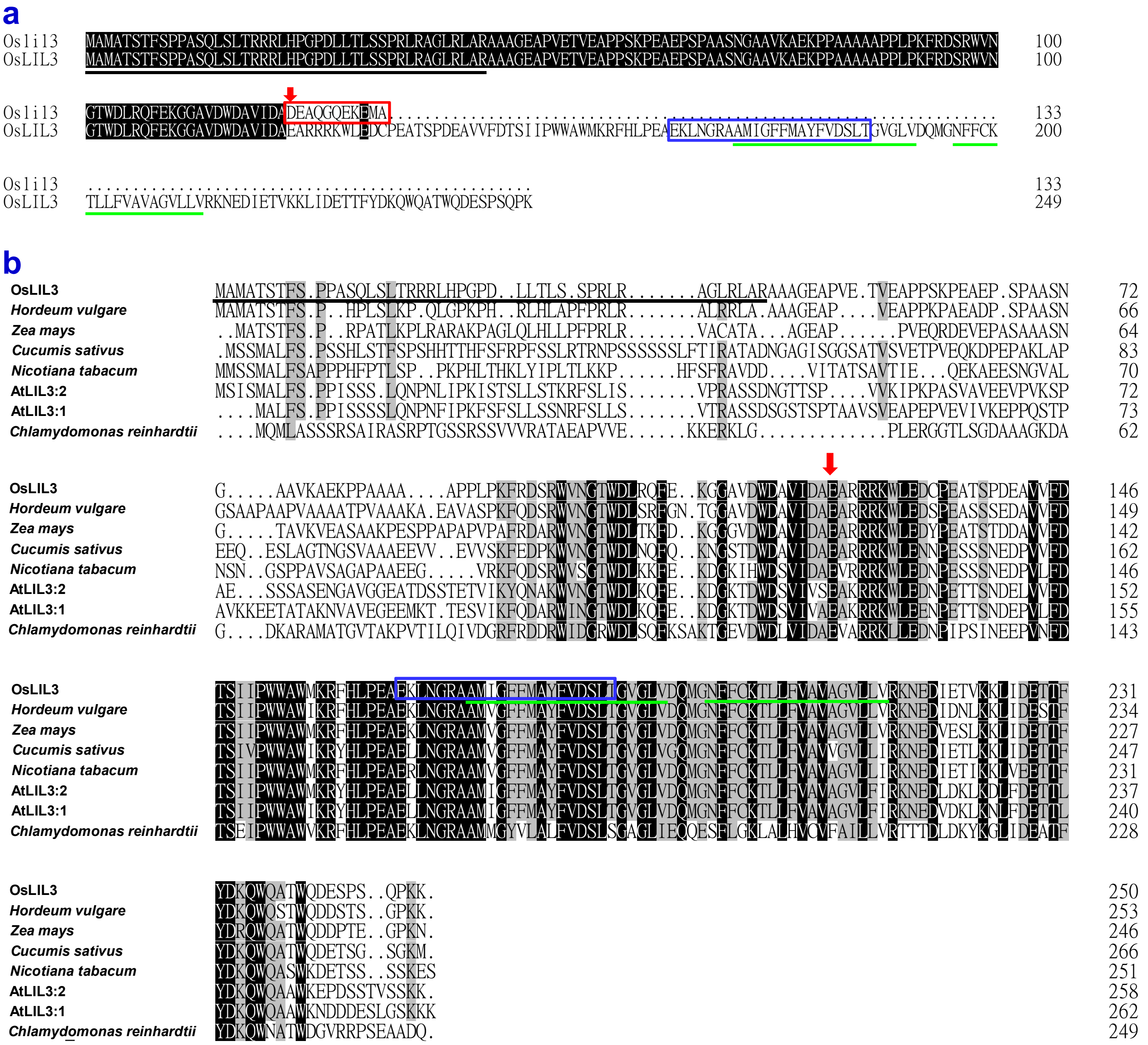

Additional file 8: Figure S4. Sequence alignment of OsLIL3 and its homologues. Identical residues are boxed in black, and similar residues (≥75% identical) are highlighted in gray. A black underline indicates the putative chloroplast signal peptides, a red arrow shows the mutant site of *637ys*, and green underlines represent the transmembrane helices predicted by HMMTOP. The blue box shows the LHC motif based on the sequence of LHC motif. The red box shows the 11 new amino acids in the mutant Oslil3 protein. GenBank accession numbers for the respective protein sequences are as Fig. 5.
