# Supplementary figures and images for: A lil3 chlp double mutant with exclusive accumulation of geranylgeranyl chlorophyll displays a lethal phenotype in rice
Source: BMC Plant Biol. 2019 Oct 29;19:456. doi: 10.1186/s12870-019-2028-z (PMC6819399; doi:10.1186/s12870-019-2028-z)

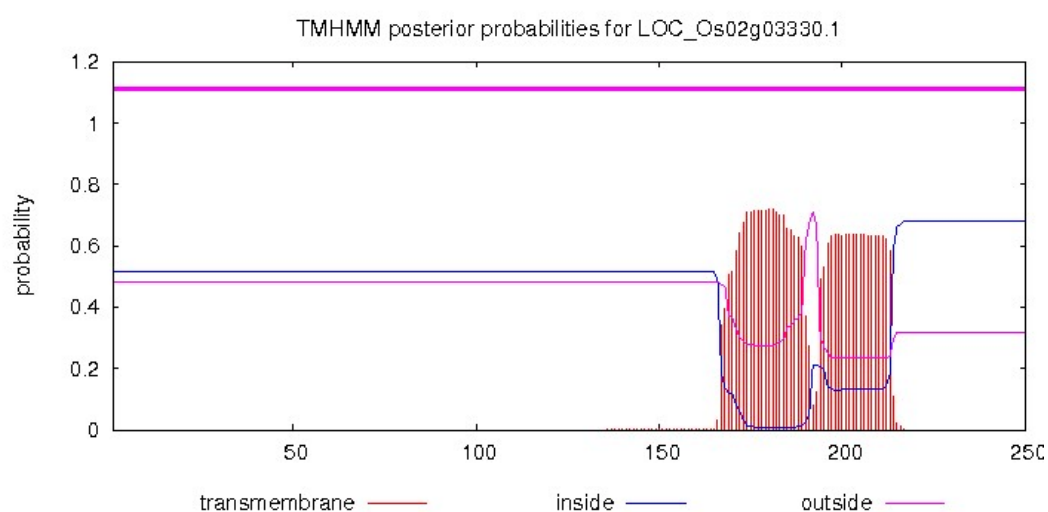

**Additional file 9: Figure S5.** Predicted transmembrane domain of the OsLIL3 (LOC\_Os02g03330) protein.

Supplement: Supplementary file 9 — Additional file 9: Figure S5. Predicted transmembrane domain of the OsLIL3 (LOC_Os02g03330) protein. (PDF 201 kb) [file 12870_2019_2028_MOESM9_ESM.pdf]
