# Supplementary material for: A lil3 chlp double mutant with exclusive accumulation of geranylgeranyl chlorophyll displays a lethal phenotype in rice
Source: BMC Plant Biol. 2019 Oct 29;19:456. doi: 10.1186/s12870-019-2028-z (PMC6819399; doi:10.1186/s12870-019-2028-z)
Supplement: Supplementary file 10 — Additional file 10: Figure S6. Ultrastructure of mesophyll cells (a) and chloroplasts (b) in the 637ys 502ys double mutant. Bars = 1 μm. (PDF 563 kb) [file 12870_2019_2028_MOESM10_ESM.pdf]

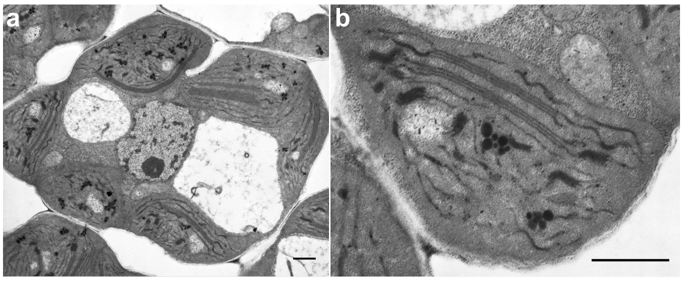

Additional file 10: Figure S6. Ultrastructure of mesophyll cells **(a)** and chloroplasts **(b)** in the *637ys 502ys* double mutant. Bars = 1 μm.
