# Supplementary material for: A lil3 chlp double mutant with exclusive accumulation of geranylgeranyl chlorophyll displays a lethal phenotype in rice
Source: BMC Plant Biol. 2019 Oct 29;19:456. doi: 10.1186/s12870-019-2028-z (PMC6819399; doi:10.1186/s12870-019-2028-z)
Supplement: Supplementary file 11 — Additional file 11: Figure S7. Phenotypic comparison and Chl composition analysis of the wild-type ZH11 and Nipponbare (NP), the 637ys mutant, the 502ys mutant and the 637ys 502ys double mutant (DM) at the three-leaf stage grown in a growth chamber under low light at constant 23 °C. (a) Phenotypic comparison. (b), (c), (d) and (e) The elution profiles of Chls in wild type, 637ys, 502ys and DM, respectively. Peaks 2, 3, 4, and 5 represent Chlphy a, ChlTHGG a, ChlDHGG a, and ChlGG a, respectively. Peaks 1, 6 and 7 represent Chlphy b, ChlDHGG b, and ChlGG b, respectively. The absorption spectra of elution profiles in acetone are the same as those in Fig. S1c and d. (PDF 546 kb) [file 12870_2019_2028_MOESM11_ESM.pdf]

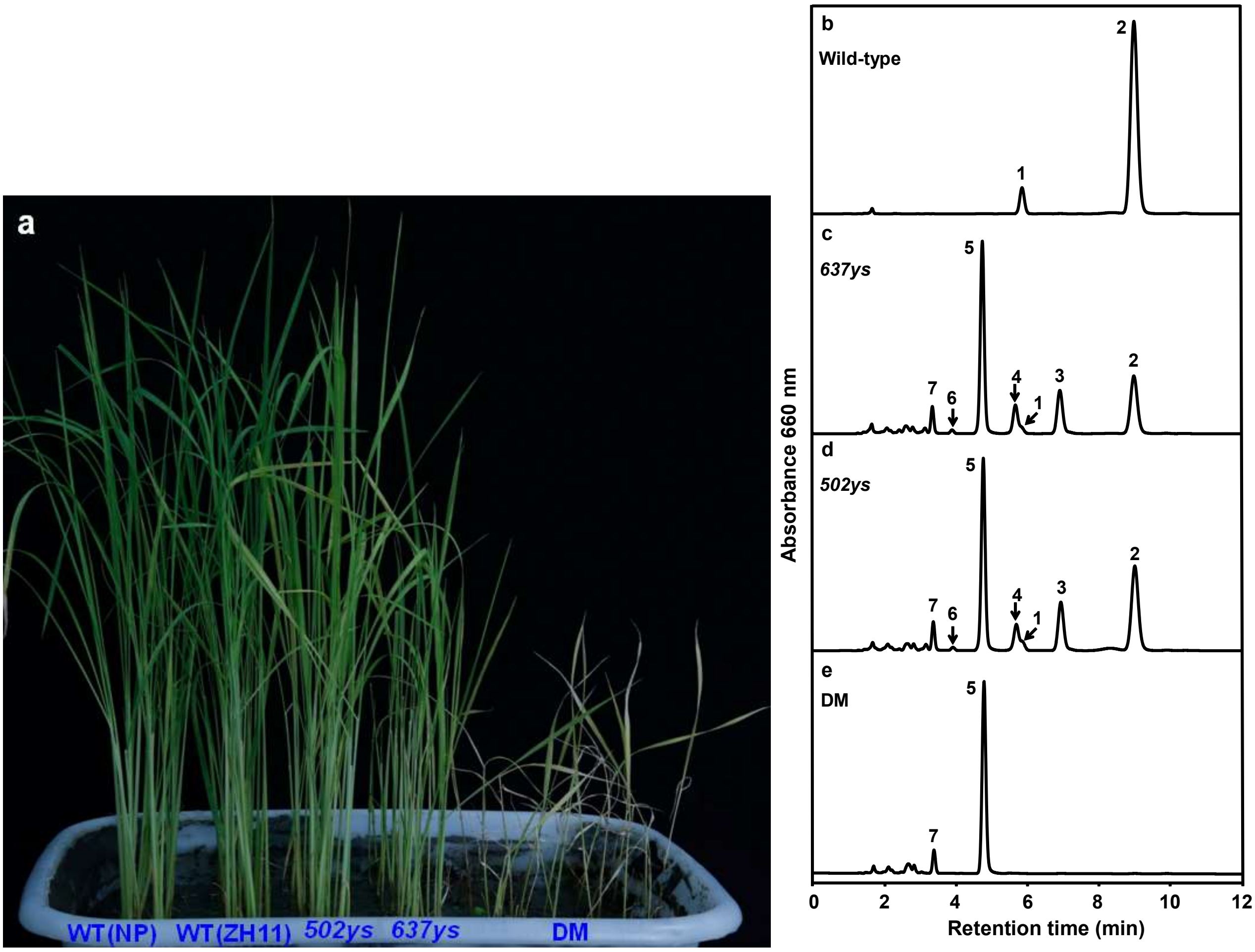

Additional file 11: Figure S7. Phenotypic comparison and Chl composition analysis of the wild-type ZH11 and Nipponbare (NP), the *637ys* mutant, the *502ys* mutant and the *637ys 502ys* double mutant (DM) at the three-leaf stage grown in a growth chamber under low light at constant 23 °C. **(a)** Phenotypic comparison. **(b)**, **(c)**, **(d)** and **(e)** The elution profiles of Chls in wild type, *637ys*, *502ys* and DM, respectively. Peaks 2, 3, 4, and 5 represent Chl_{phy} *a*, Chl_{THGG} *a*, Chl_{DHGG} *a*, and Chl_{GG} *a*, respectively. Peaks 1, 6 and 7 represent Chl_{phy} *b*, Chl_{DHGG} *b*, and Chl_{GG} *b*, respectively. The absorption spectra of elution profiles in acetone are the same as those in **Figure S1c** and **d**.
