# Supplementary material for: A lil3 chlp double mutant with exclusive accumulation of geranylgeranyl chlorophyll displays a lethal phenotype in rice
Source: BMC Plant Biol. 2019 Oct 29;19:456. doi: 10.1186/s12870-019-2028-z (PMC6819399; doi:10.1186/s12870-019-2028-z)
Supplement: Supplementary file 12 — Additional file 12: Figure S8. Total Chl contents in 502ys and its wild-type (WT) grown under low light (LL) or high light (HL) at constant temperature (23 °C or 30 °C), in mg g fresh weight− 1. Data are shown as mean ± SD. Error bars represent standard deviations of three independent biological replicates. Asterisks indicate statistically significant differences compared with the wild-type at P < 0.01. (PDF 560 kb) [file 12870_2019_2028_MOESM12_ESM.pdf]

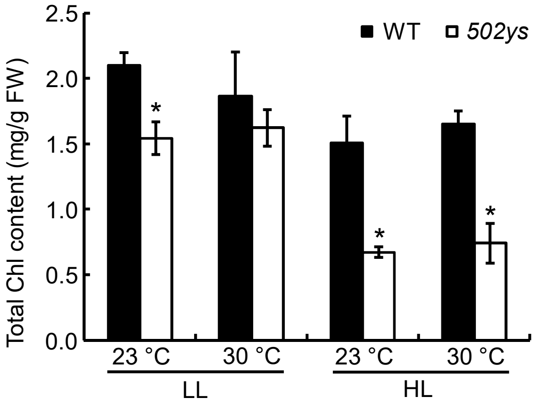

Additional file 12: Figure S8. Total Chl contents in *502ys* and its wild-type (WT) grown under low light (LL) or high light (HL) at constant temperature (23 °C or 30 °C), in mg g fresh weight^{-1}. Data are shown as mean ± SD. Error bars represent standard deviations of three independent biological replicates. Asterisks indicate statistically significant differences compared with the wild-type at $P < 0.01$.
