# Supplementary material for: A lil3 chlp double mutant with exclusive accumulation of geranylgeranyl chlorophyll displays a lethal phenotype in rice
Source: BMC Plant Biol. 2019 Oct 29;19:456. doi: 10.1186/s12870-019-2028-z (PMC6819399; doi:10.1186/s12870-019-2028-z)
Supplement: Supplementary file 13 — Additional file 13: Figure S9 Analysis of vitamin E in leaves and grains of 502ys. Elution profiles of the tocopherol standards (a), tocopherols in leaves of wild-type Nipponbare (b) and 502ys (c), tocopherols and tocotrienols in grains of Nipponbare (d) and 502ys (e) were detected by fluorescence with excitation at 290 nm and emission at 330 nm. (f) Tocopherol contents in leaves and grains of Nipponbare and 502ys were quantified by using tocopherol standards. (g) The peak area of tocotrienols in grains of Nipponbare (WT) and 502ys. α-T, α-tocopherol; γ-T, γ-tocopherol. The tocopherol standards were prepared as described in Fig. 5. α-T3, γ-T3 and δ-T3 represent α-tocotrienol, γ-tocotrienol and δ-tocotrienol, respectively. Peaks 1 and 2 represent α-tocopherol and γ-tocopherol; Peak 3 is δ-tocopherol which does not exist in rice and was used as control. Peaks 4, 5 and 6 represent α-tocotrienol, γ-tocotrienol and δ-tocotrienol, respectively. Peak 7 might be the isomer of γ-tocopherol. Error bars represent standard errors of three independent biological replicates. Asterisks indicate statistically significant differences compared with the wild-type at P < 0.01. (PDF 758 kb) [file 12870_2019_2028_MOESM13_ESM.pdf]

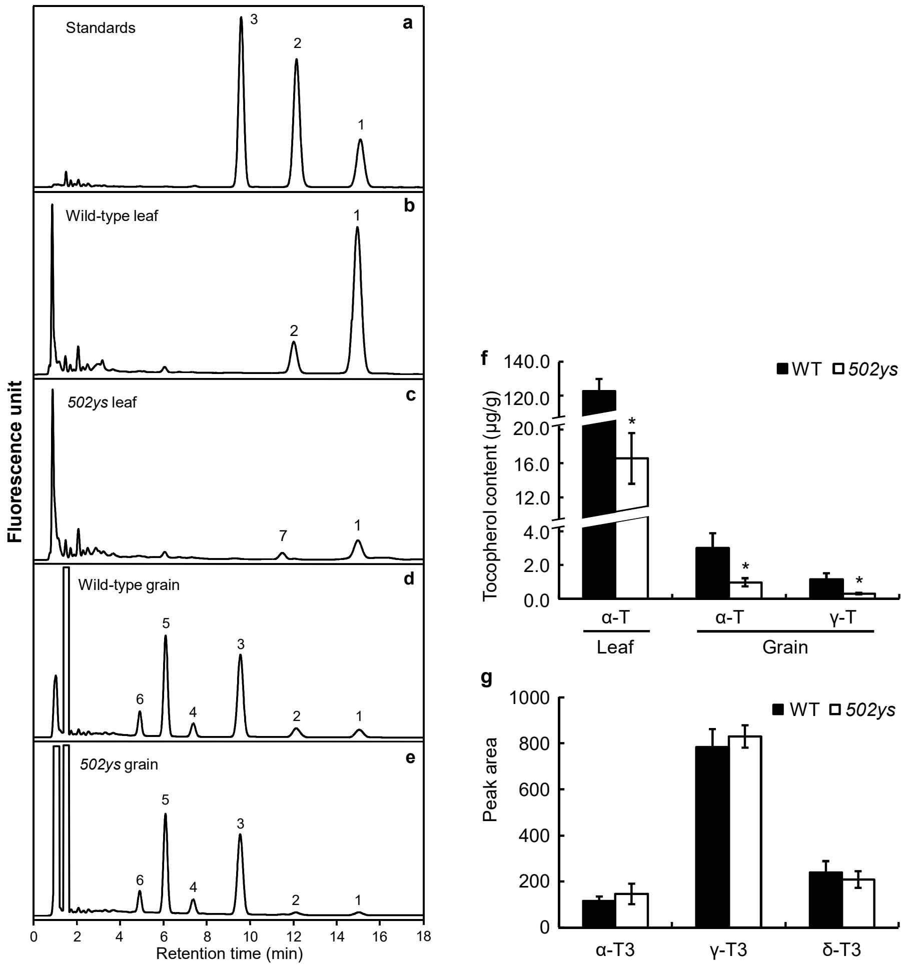

Additional file 13: Figure S9. Analysis of vitamin E in leaves and grains of *502ys*. Elution profiles of the tocopherol standards **(a)**, tocopherols in leaves of wild-type Nipponbare **(b)** and *502ys* **(c)**, tocopherols and tocotrienols in grains of Nipponbare **(d)** and *502ys* **(e)** were detected by fluorescence with excitation at 290 nm and emission at 330 nm. **(f)** Tocopherol contents in leaves and grains of Nipponbare and *502ys* were quantified by using tocopherol standards. **(g)** The peak area of tocotrienols in grains of Nipponbare (WT) and *502ys*. α-T, α-tocopherol; γ-T, γ-tocopherol. The tocopherol standards were prepared as described in Figure 5. α-T3, γ-T3 and δ-T3 represent α-tocotrienol, γ-tocotrienol and δ-tocotrienol, respectively. Peaks 1 and 2 represent α-tocopherol and γ-tocopherol; Peak 3 is δ-tocopherol which doesn't exist in rice and was used as control. Peaks 4, 5 and 6 represent α-tocotrienol, γ-tocotrienol and δ-tocotrienol, respectively. Peak 7 might be the isomer of γ-tocopherol. Error bars represent standard errors of three independent biological replicates. Asterisks indicate statistically significant differences compared with the wild-type at $P < 0.01$.
