# Supplementary material for: A lil3 chlp double mutant with exclusive accumulation of geranylgeranyl chlorophyll displays a lethal phenotype in rice
Source: BMC Plant Biol. 2019 Oct 29;19:456. doi: 10.1186/s12870-019-2028-z (PMC6819399; doi:10.1186/s12870-019-2028-z)
Supplement: Supplementary file 14 — Additional file 14: Figure S10. Expression analysis of genes involved in photosynthesis and Chl biosynthesis in 502ys. Actin 1 was amplified as an internal reference. The expression level of each gene in wild types was set to 1.0, and those in 637ys and 502ys mutants were calculated accordingly. Error bars represent standard errors of three independent biological replicates. The asterisk indicates statistically significant differences compared with the wild-type at P < 0.05. (PDF 591 kb) [file 12870_2019_2028_MOESM14_ESM.pdf]

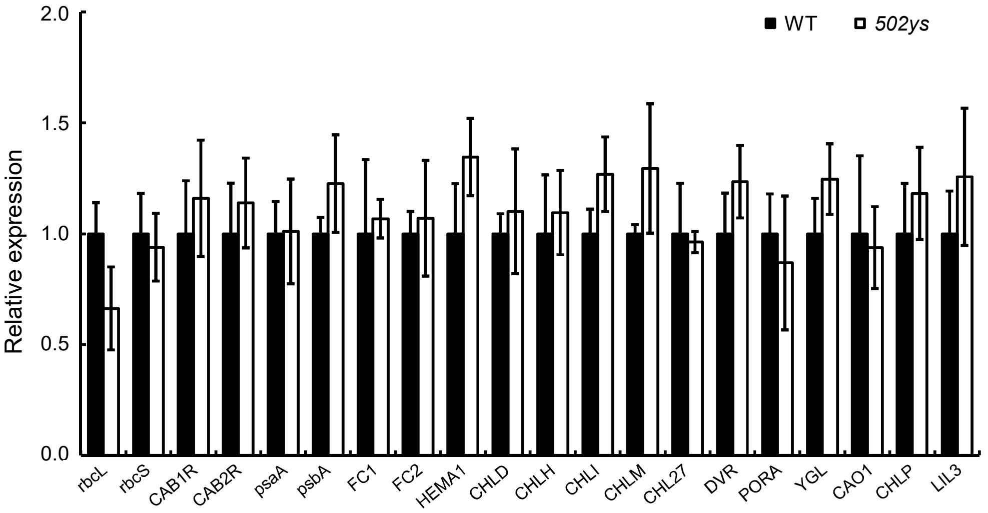

Additional file 14: Figure S10. Expression analysis of genes involved in photosynthesis and Chl biosynthesis in *502ys*. *Actin 1* was amplified as an internal reference. The expression level of each gene in wild types was set to 1.0, and those in *637ys* and *502ys* mutants were calculated accordingly. Error bars represent standard errors of three independent biological replicates. Asterisk indicates statistically significant differences compared with the wild-type at $P < 0.05$.
