# Supplementary material for: A lil3 chlp double mutant with exclusive accumulation of geranylgeranyl chlorophyll displays a lethal phenotype in rice
Source: BMC Plant Biol. 2019 Oct 29;19:456. doi: 10.1186/s12870-019-2028-z (PMC6819399; doi:10.1186/s12870-019-2028-z)
Supplement: Supplementary file 15 — Additional file 15: Figure S11. Expression patterns of OsCHLP gene. The expression levels were determined by real-time PCR in root (R), and leaf (L), leaf sheath (LS), panicle (P), and stem (S) of wild type grown in a paddy field. The levels of transcripts were normalized to rice Actin 1 gene as an internal control. Error bars represent standard errors of three independent biological replicates. (PDF 569 kb) [file 12870_2019_2028_MOESM15_ESM.pdf]

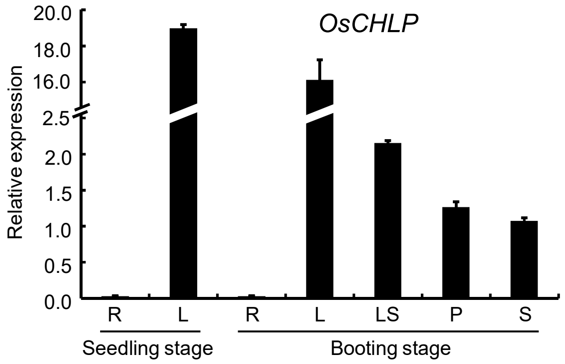

Additional file 15: Figure S11. Expression patterns of *OsCHLP* gene. The expression levels were determined by real-time PCR in root (R) and leaf (L), leaf sheath (LS), panicle (P), and stem (S) of wild type grown in a paddy field. The levels of transcripts were normalized to rice *Actin 1* gene as an internal control. Error bars represent standard errors of three independent biological replicates.
