# Supplementary material for: A lil3 chlp double mutant with exclusive accumulation of geranylgeranyl chlorophyll displays a lethal phenotype in rice
Source: BMC Plant Biol. 2019 Oct 29;19:456. doi: 10.1186/s12870-019-2028-z (PMC6819399; doi:10.1186/s12870-019-2028-z)
Supplement: Supplementary file 16 — Additional file 16: Table S5. Primers used in RT-PCR. (PDF 277 kb) [file 12870_2019_2028_MOESM16_ESM.pdf]

Additional file 16: Table S5 Primers used in RT-PCR

Gene name	Forward primer (5′–3′)	Reverse primer (5′–3′)	Reference
rbcL	CTTGGCAGCATTCCGAGTAA	ACAACGGGCTCGATGTGATA	Su et al., 2012
rbcS	CAGCAATGGCGGCAGGAT	AGGGCACCCACTTGGAACG	Li et al., 2015
CAB1R	AGATGGGTTTAGTGCGACGAG	TTTGGGATCGAGGGAGTATTT	Su et al., 2012
CAB2R	TGTTCTCCATGTTCGGCTTCT	GCCCAGGCGTTGTTGTTGA	Su et al., 2012, Ma et al. 2017
psaA	GGAGGTGGCGAGTTAGTA	GATTTGCTTTATCGGGTAT	Li et al., 2015
psbA	TATGGGTCGTGAGTGGGA	TTATGCTCTGCCTGGAAT	Li et al., 2015
FC1	GGTCAACAGGGTGTAAAGAG	GTGCATCCAAGAGCTGGAAC	Inagaki et al., 2015
FC2	CTTGCCTTATGTTGGTGCTA	AGCCCCACTCCCATACAGTC	Inagaki et al., 2015
HEMA1	CGCTATTTCTGATGCTATGGGT	TCTTGGGTGATGATTGTTTGG	Su et al., 2012
CHLD	CAAGGGTCGCCCAAGGTAAA	CTTCAGGTCCGAGAGTGCAG	
CHLH	CCAATCCGTAACCCGAAGGT	CAATAATTTTGGCGCTCTTCAA	Inagaki et al., 2015
CHLI	TTCGACAGGGATCCAAAGGC	ACAGCACCAAGGTTACTCCG	
CHLM	GGCTTCATCTCCACGCAGTT	GACGAATCGAAGACGCACAA	
CHL27	CCGCAGGCTCAAGAAAAC	ATCAGACAGCCCCTTGTTCA	Inagaki et al., 2015
DVR	CCATTGCCAGTTTCTTGGTG	AATTGAATGGCTAATGGCGT	Inagaki et al., 2015
PORA	TGTACTGGAGCTGGAACAACAA	GAGCACAGCAAAATCCTAGACG	Su et al., 2012
YGL	GGCACTGCTAGGACTCAC	CCCAAGACGAAGAACGGT	Wu et al., 2007
CAO1	GATCCATACCCGATCGACAT	CGAGAGACATCCGGTAGAGC	Su et al., 2012
CHLP	GACCGCGATCGAACACTTTG	TTGATCAGCCGCGAATCTGT	
LIL3	GCAGCCCTTTTCTCAACCCA	AACCAAGTAACCCGCAGCAA	
Actin 1	TGTATGCCAGTGGTCGTACCA	CCAGCAAGGTCGAGACGAA	Li et al., 2015

Inkagai, N., Kinoshita, K., Kagawa, T., Tanaka, A., Ueno, O., Shimada, H., et al. (2015). Phytochrome B mediates the regulation of chlorophyll biosynthesis through transcriptional regulation of *ChlH* and *GUN4* in rice seedlings. *PLoS ONE* 10: e0135408. doi: 10.1371/journal.pone.0135408

Li, C.M., Hu, Y., Huang, R., Ma, X.Z., Wang, Y., Liao, T.T., et al. (2015). Mutation of *FdC2* gene encoding a ferreoxin-like protein with C-terminal extension causes yellow-green leaf phenotype in rice. *Plant Sci.* 238, 127–134. doi: 10.1016/j.plantsci.2015.06.010

Ma, X., Sun, X., Li, C., Huan, R., Sun, C., Wang, Y., et al. (2017). Map-based cloning and characterization of the novel yellow-green leaf gene ys83 in rice (*Oryza sativa*). *Plant Physiol. Biochem.* 111, 1–9. doi: 10.1016/j.plaphy.2016.11.007

Su, N., Hu, M.L., Wu, D.X., Wu, F.Q., Fei, G.L., Lan, Y., et al. (2012). Disruption of a rice pentatricopeptide repeat protein causes a seedling-specific albino phenotype and its utilization to enhance seed purity in hybrid rice production. *Plant Physiol.* 159, 227–238. doi: 10.1104/pp.112.195081

Wu, Z.M., Zhang, X., He, B., Diao, L.P., Sheng, S.L., Wang, J.L., et al. (2007). A chlorophyll-deficient rice mutant with impaired chlorophyllide esterification in chlorophyll biosynthesis. *Plant Physiol.* 145, 29–40. doi: 10.1104/pp.107.100321
